# Classical cannabinoid receptors as target in cancer-induced bone pain: a systematic review, meta-analysis and bioinformatics validation

**DOI:** 10.1038/s41598-024-56220-0

**Published:** 2024-03-09

**Authors:** Feier Zeng, Abbie Wade, Kade Harbert, Shrina Patel, Joshua S. Holley, Cornelia K. Dehghanpuor, Thomas Hopwood, Silvia Marino, Antonia Sophocleous, Aymen I. Idris

**Affiliations:** 1 Department of Oncology and Metabolism, University of Sheffield, Medical School, Beech Hill Road, Sheffield, S10 2RX UK; 2 Department of Physiology and Cell Biology, University of Arkansas for Medical Sciences (UAMS), BioMed II, 238-2, Little Rock, AR USA; 3 Department of Life Sciences, School of Sciences, European University Cyprus, 6 Diogenes Street, 1516 Nicosia, Cyprus

**Keywords:** CB_1_, CB_2_, Pain, Cancer, Animal models, Bone, Preclinical, Cancer therapy, Computational biology and bioinformatics, Drug discovery

## Abstract

To test the hypothesis that genetic and pharmacological modulation of the classical cannabinoid type 1 (CB_1_) and 2 (CB_2_) receptors attenuate cancer-induced bone pain, we searched Medline, Web of Science and Scopus for relevant skeletal and non-skeletal cancer studies from inception to July 28, 2022. We identified 29 animal and 35 human studies. In mice, a meta-analysis of pooled studies showed that treatment of osteolysis-bearing males with the endocannabinoids AEA and 2-AG (mean difference [MD] − 24.83, 95% confidence interval [^95%^CI] − 34.89, − 14.76, *p* < 0.00001) or the synthetic cannabinoid (CB) agonists ACPA, WIN55,212-2, CP55,940 (CB_1/2_-non-selective) and AM1241 (CB_2_-selective) (MD − 28.73, ^95%^CI − 45.43, − 12.02, *p* = 0.0008) are associated with significant reduction in paw withdrawal frequency. Consistently, the synthetic agonists AM1241 and JWH015 (CB_2_-selective) increased paw withdrawal threshold (MD 0.89, ^95%^CI 0.79, 0.99, *p* < 0.00001), and ACEA (CB_1_-selective), AM1241 and JWH015 (CB_2_-selective) reduced spontaneous flinches (MD − 4.85, ^95%^CI − 6.74, − 2.96, *p* < 0. 00001) in osteolysis-bearing male mice. In rats, significant increase in paw withdrawal threshold is associated with the administration of ACEA and WIN55,212-2 (CB_1/2_-non-selective), JWH015 and AM1241 (CB_2_-selective) in osteolysis-bearing females (MD 8.18, ^95%^CI 6.14, 10.21, *p* < 0.00001), and treatment with AM1241 (CB_2_-selective) increased paw withdrawal thermal latency in males (mean difference [MD]: 3.94, ^95%^CI 2.13, 5.75, *p* < 0.0001), confirming the analgesic capabilities of CB_1/2_ ligands in rodents. In human, treatment of cancer patients with medical cannabis (standardized MD − 0.19, ^95%^CI − 0.35, − 0.02, *p* = 0.03) and the plant-derived delta-9-THC (20 mg) (MD 3.29, CI 2.24, 4.33, *p* < 0.00001) or its synthetic derivative NIB (4 mg) (MD 2.55, ^95%^CI 1.58, 3.51, *p* < 0.00001) are associated with reduction in pain intensity. Bioinformatics validation of KEGG, GO and MPO pathway, function and process enrichment analysis of mouse, rat and human data revealed that CB_1_ and CB_2_ receptors are enriched in a cocktail of nociceptive and sensory perception, inflammatory, immune-modulatory, and cancer pathways. Thus, we cautiously conclude that pharmacological modulators of CB_1/2_ receptors show promise in the treatment of cancer-induced bone pain, however further assessment of their effects on bone pain in genetically engineered animal models and cancer patients is warranted.

## Introduction

Pain is a serious complication of advanced cancer^1–4^. A large proportion of cancer survivors suffer from acute and chronic pain caused by the disease progression and/or as a result of treatment side-effect^5^. Bone pain is a debilitating aspect of primary bone carcinomas such as osteosarcoma and multiple myeloma^6–10^, but its prevalence is increasingly common among long-term survivors of metastatic cancers^6,7,10–14^. Although advances in early detection and treatments are effective in alleviating cancer related skeletal events, bone pain is difficult to treat, and resistant to conventional analgesics such as non-steroidal anti-inflammatory drugs (NSAIDs) and opiate^1,12,15–18^. As cancer survivability increases^5^, there is an expectation that bone pain may become a significant contributor to the burden of metastatic cancer^15,17,19–21^. Thus, there is a need to explore the analgesic efficacy of multi-modal and multi-target therapies such as cannabinoids.

The endogenous cannabinoid (endocannabinoid) system of ligands, receptors, and enzymes is implicated in pain perception, and it is known to regulate a plethora of biological processes and disorders including inflammation, immunity, and cancer^22–31^. In the skeleton, the classical type 1 (CB_1_) and/or 2 (CB_2_) cannabinoid receptors are expressed by peripheral neurons, microglia, and immune and bone cells, particularly osteoblasts, osteoclasts, osteocytes, and bone-marrow derived adipocytes^32–41^. A number of studies, including ours^40,42,43^, have reported that genetic and pharmacological modulation of CB_1_ and/or CB_2_ receptors both enhance and reduce bone cell activity and remodelling in health, ageing, and disease^33–39^. The drive to legalise preparations of the plant-derived delta-9-tetrahydrocannabinol (delta-9-THC) coupled with the increase in the commercialization of the non-psychoactive cannabidiol (CBD) have accelerated their availability and on-/off-label use^44–49^. In similar manner to the endocannabinoids 2-arachidonoylglycerol (2-AG) and *N*-Arachidonoylethanolamine (anandamide, AEA), CBD and delta-9-THC activate and regulate the activity of the classical CB_1_ and CB_2_ receptors. Furthermore, they individually or together in the clinical mixture Sativex® (aka Nabiximols®) affect the initiation and progression pain associated with inflammation, cancer and/or chemotherapy—alone or in the combination with opiates^50–55^. In recent years, there has been an explosion in animal and human studies that reported the nociception properties of various natural and synthetic cannabinoids- thereby indicating potential therapeutic opportunities^6,7,16,56,57^.

Endogenous and most plant-derived and synthetic cannabinoids tested thus far are known to bind, activate, and/or indirectly influence signalling transduction pathways downstream of the classical CB_1_ and CB_2_ receptors—albeit with different degrees of selectivity^58–60^. Owing to the multi-factorial nature of bone—tumour—sensory nerve cell interactions and crosstalk in the skeleton^19,20,61–63^ and disparity in receptor expression among these cells, there is an urgent need for examining of the mechanism(s) by which CB_1/2_ receptor ligands affect the initiation and progression of bone pain, and understanding of the downstream signal transduction pathways and biological and pathological functions and processes involved^64–71^. With this in mind, we took the decision to conduct a combined systematic review, meta-analysis, and bioinformatics validation of skeletal (and non-skeletal) studies to interrogate the hypothesis that genetic and pharmacological modulation of the classical CB_1_, CB_2_ receptors or both are associated with significant reduction in cancer-induced pain, including bone pain, in animal models and humans.

## Materials and methods

### Meta-analysis

We conducted the systematic review and meta-analysis according to the Preferred Reporting Items for Systematic Reviews and Meta-analyses (PRISMA) statement^72^.

### Data sources and search strategy

PubMed, Web of Science, and Scopus were systematically searched in July 2022 for articles featuring a list of keywords related to three broad categories, namely the endocannabinoid system, cancer, and pain (Table S1). Search results were limited to articles that reported human and animal studies, amalgamated into an EndNote 20 (Clarivate, UK) library.

### Inclusion and exclusion criteria

Studies that used animal models to assess the effects of pharmacological and/or genetic manipulation of the classical cannabinoid CB_1_ and/or CB_2_ receptors using standard rodent cancer models of mechanical allodynia, thermal hyperalgesia, and spontaneous and ambulatory behaviour assays^6,73^ (Table 1) and human studies that measured the effect of CB_1/2_ natural and synthetic ligands on intensity of cancer-induced pain^2,3^ were included (Table S1). Additionally, studies were included if reported the pharmacological effects of natural endocannabinoids or plant-derived (*Cannabis sativa*) or synthetic verified cannabinoid receptor ligands (refer to types of *intervention* below). Excluded studies include case reports, reviews, conference papers/abstracts, commentaries and editorial pieces, articles published in a language other than English, and studies that failed to meet the inclusion criteria.

**Table 1:** Summary of the number and characteristics of animal and human studies included in this systematic review.

In vivo studies	Human studies
Intervention/modification	Intervention
Pharmacological manipulation (29)	Medical cannabis (15)
Endocannabinoids (2)	Regular cannabis use (5)
Synthetic agonists (22)	THC/CBD (4)
Synthetic antagonists/inverse agonists (5)	THC (3)
Genetic manipulation (0)	Sativex (Nabiximols) (3)
Species/strain	Nabilone (2)
Rat	Benzopyranoperidine (1)
Sprague Dawley (5)	NIB (nitrogen analogue of THC) (1)
Wistar (7)	Water-wash processed *Cannabis sativa* L. leaves powder (1)
Mouse	Study types
C3H^+^ (16)	Prospective studies (21)
BALB/c (1)	RCT (8)
Sex	Crossover (6)
Female (6)	Quasi-experimental (2)
Male (23)	Cohort (5)
Model types	Retrospective (10)
Rat (13)	Observational cross-sectional (4)
Walker 256 Mammary Gland Carcinoma Cells (10)	Outcomes
Mammary MRMT-1 Gland Carcinoma (3)	Numeric rating scale (NRS) (8)
Mouse (16)	Edmonton symptom assessment scale (ESAS) (6)
Fibrosarcoma cells (NCTC 2472) (15)	Visual analogue scale (VAS) (2)
4T1 cells (1)	Pain symptom scale 0–100 (2)
Outcomes	Pain reduction (2)
Mechanical allodynia (25)	Pain intensity (2)
Paw withdrawal frequency (6)	Non-specified pain scale of 0–10 (2)
Paw withdrawal threshold (18)	Assessment of Quality of Life-8 dimensions (AQoL-8D) pain analysis (1)
Forelimb grip force (1)	FACES Pain Rating Scale for pain (1)
Thermal hyperalgesia assay (9)*	No reference to pain scale (9)
Paw withdrawal thermal latency (9)	
Spontaneous / ambulatory behaviour (6)^**£**^	
Spontaneous flinching (4)	
Ambulatory score (2)	

*Indicates that 7 of the included 9 articles reported mechanical allodynia and spontaneous/ambulatory behaviour.

^£^Represent 4 of 6 included articles also reported mechanical allodynia and thermal hyperalgesia assay.

^+^Denotes C3H strains used include He, HeN, HeJ, HeNCr MTV-.

### Types of intervention

The systematic review and meta-analysis include natural and synthetic cannabinoid receptor ligands that have been verified to selectively bind to, interact, and/or activate signalling pathways downstream of the classical CB_1_ and/or CB_2_ receptors^60,64–71^. Natural and plant-derived ligands include the CB_1/2_-non-selective cannabinoids delta-9-tetrahydrocannabinol (delta-9-THC) and cannabidiol (CBD), and endocannabinoids including 2-arachidonoylglycerol (2-AG) and *N*-Arachidonoylethanolamine (anandamide, AEA). Synthetic CB_1/2_ ligands were classified into verified agonists and antagonists/inverse agonists as previously described^60^. The list of agonists includes (but not limited to) the CB_1/2_-non-selective WIN 55,212-2 and ACPA, CB_1_-selective JWH015 and CP55,940, CB_2_-selective JWH133 and AM1241. The list of antagonists/inverse agonists includes (but not limited to) the CB_1_-selective AM251 and SR141716A (Rimonabant), and the CB_2_-selective AM630, AM281 and SR144528. We also included the nitrogen-containing benzopyran derivative, modification of delta-1-trans-tetrahydrocannabinol (NIB)^74^. The chemical structures of the aforementioned cannabinoid ligands are shown in Supplementary Fig. S4.

### Effect parameters

We included studies assessing the effects of natural and synthetic verified ligands of CB_1/2_ on a panel of standard experimental outcomes in animal (in vivo*,* Tables S2) models of cancer, and cancer patients (human, Tables S3). Outcomes from included animal studies includes (but not limited to) cancer-induced changes in paw withdrawal frequency (%), threshold (g) and latency (s), number of spontaneous flinches and score of ambulatory activity (Tables S2). Outcomes from studies in cancer patients include pain intensity scores using the following scales (but not limited to): Numeric Rating Scale (NRS; 0–10), Edmonton Symptom Assessment Scale (ESAS; 0–10), Visual Analogue Scale (VAS; continuum; no pain–worst pain), Pain symptom scale (0–100), and the FACES Pain Rating Scale for pain (Tables S3).

### Data collection and analysis

Article selection, review and assessment was performed by at least two independent researchers, and any conflicts were resolved by referral to a third researcher. Mean and standard deviation (SD) or standard error measurement (SEM) were extracted from original figures using the online tool WebPlotDigitizer (https://apps.automeris.io/wpd/). If SD was not available this was calculated from SE using the following formula: SD = SE × SQRT(n). If SE was not available this was calculated from 95% CI using the following formula: SE = (upper limit − lower limit)/3.92. The mean difference (MD) of 2 studies or more that deemed of the same design, outcome and unit of measure was used as the effect measure. Otherwise, standardized (std.) MD was used. Heterogeneity was determined by fixed effect analysis if effects were small to moderate (i.e. I^2^ < 50%), and by random effect analysis model if effects were considered to be high (i.e. I^2^ > 50%).

### Quality assessment

For the assessment of quality of non-randomized studies, the Syrcle risk of bias tool^75^ was used for animal studies, and the revised Cochrane risk-of-bias tool (RoB 2) for randomized trials (RoB 2 tool) or the Newcastle–Ottawa Scale (NOS)^76^ were used for human studies. Certainty of the evidence was assessed by the grading of recommendations assessment, development, and evaluation (GRADE) approach^77^. The GRADE approach was also adapted for preclinical systematic reviews as previously described^78^.

### Publication bias

The funnel plot asymmetry analysis was not performed since all pooled analyses included less than 10 studies^79^.

### Bioinformatics analysis

To identify enriched pathways, functions, processes, and diseases associated with the classical cannabinoid receptors CB_1_ and/or CB_2_, enrichment analyses in Kyoto Encyclopedia of Genes and Genomes (KEGG), Gene Ontology (GO) and Monarch Phenotype Ontology (MPO) Initiative, and DISEASES were carried out using the database of Search Tool for the Retrieval of Interacting Genes/proteins (STRING, version 11.5)^80,81^. The minimum required interaction score was high confidence (0.7), cut-off threshold was False Discovery Rate (FDR) less than 0.05^82^, and the rest of the parameters were used as defaulted. Bubble charts and Venn diagrams were generated using MATLAB (version 9.12).

## Results

### Study selection and characteristics

**Figure 1:**
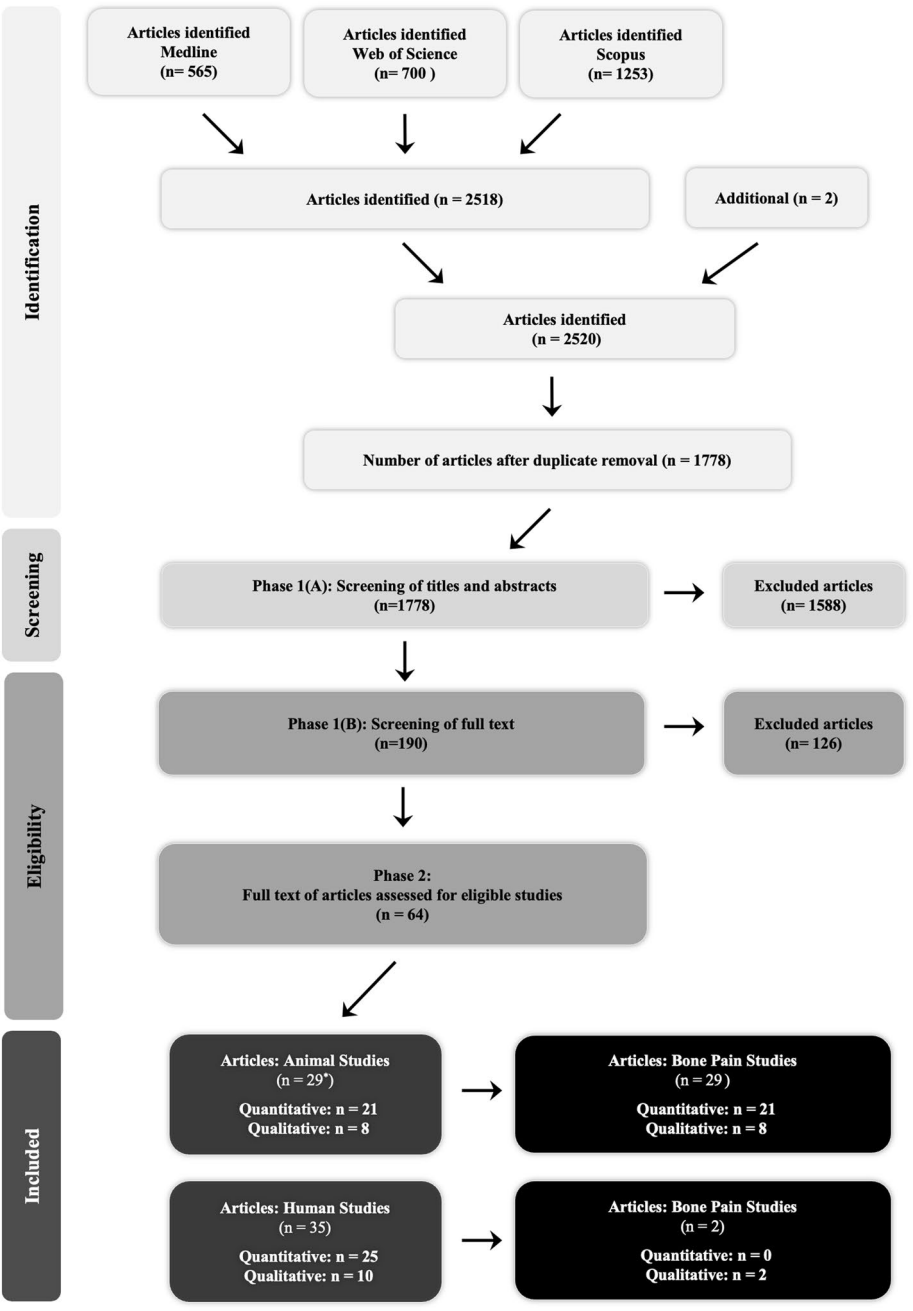
Systematic reviews and meta-analyses (PRISMA) flow diagram. n Denotes article number.

A total of 2520 English-language articles were identified, and 742 duplicate records were removed. As shown in Fig. 1, out of the remaining 1778 articles, 1588 were excluded based on title and/or abstract. A total of 190 articles were assessed for eligibility, and 126 of which were excluded. The meta-analysis included 25 human and 21 animal intervention studies, where 18 studies were included in the qualitative synthesis; 10 of which were human studies and 8 included animal experiments (Fig. 1). All of the included articles (29 animal and 35 human) were published from 1974 to 2022, and reported cancer-induced bone pain in rodents, and pain intensity in humans. Incidence of bone pain were considered in animals bearing osteolytic tumours or cancer patients that specifically measured bone pain (Fig. 1). Other characteristics of the included animal and human studies are summarised in Tables S2–S5.

### Included studies

**Figure 2:**
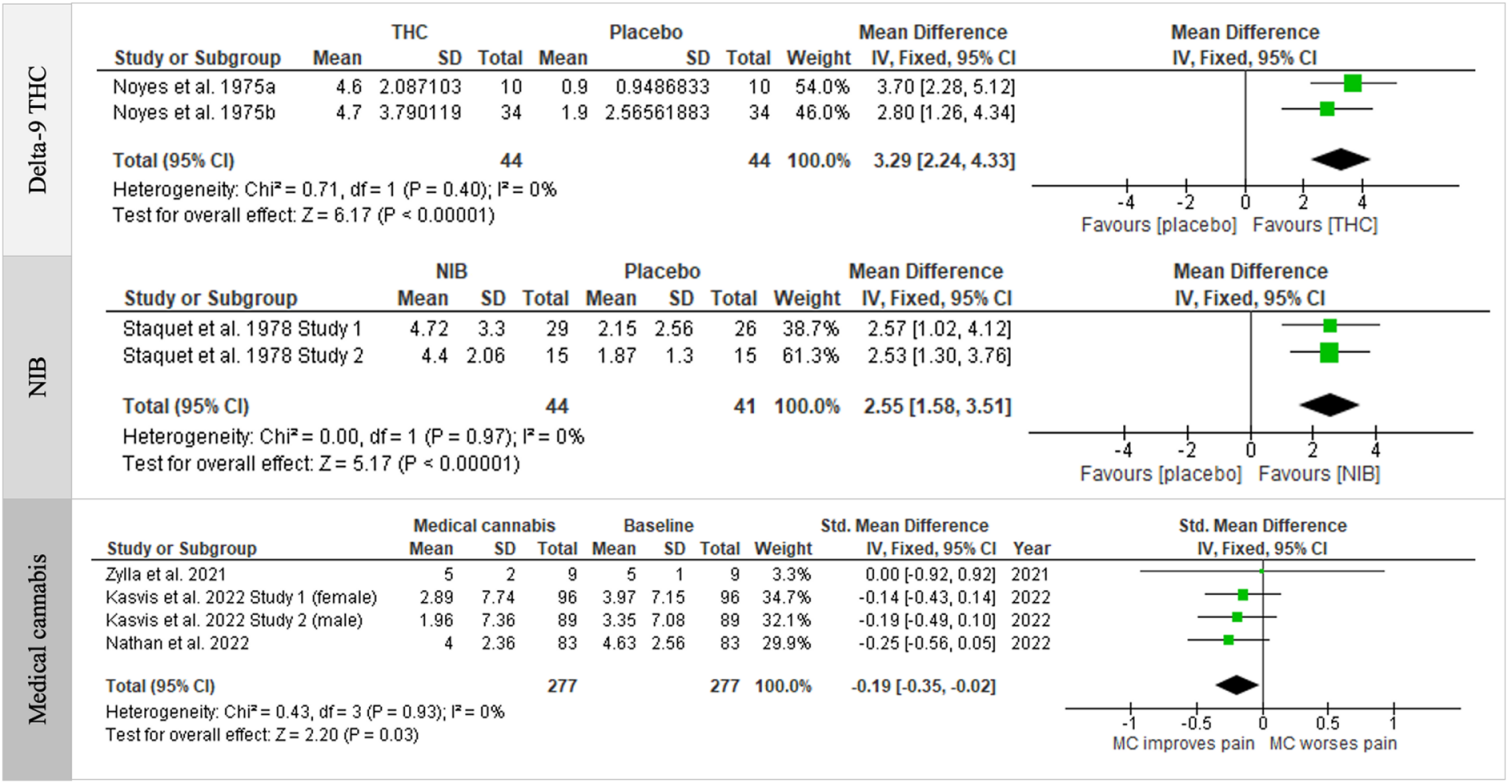
Forest plot of included human studies showing association between treatment with delta-9-tetrahydrocannabinol (delta-9-THC, 20 mg, top), its nitrogen-containing benzopyran derivative (a modification of delta-1-trans-tetrahydrocannabinol, NIB, 4 mg, middle) and medical cannabis (MC, bottom) and reduction in pain intensity in cancer patients.

Summary of meta-analysis showing significant and non-significant association of pharmacological modulation of CB_1/2_ receptors in animal models of cancer (Table 2 and S6), and in cancer patients (Fig. 2 and Table S7). Data reported in a format unsuitable for pooling or considered too heterogeneous are included in narrative synthesis (Table 3).

**Table 2:** Summary of meta-analysis showing significant association of cancer-induced pain with pharmacological modulation of CB1 and/or CB2 receptors in rodents.

Intervention	Outcome	Gender (species)	CB receptor (strain, no. studies)	Groups	Subgroup (std.) mean difference (95%CI)	Overall (std.) mean difference (95% CI)	Statistical method	Test for heterogeneity	Test for overall effect
Endo-cannabinoids	Paw withdrawal frequency (%)	Male (Mice)	Endogenous cannabinoids (C3H/HeNCr MTV-, 1) Endogenous cannabinoids (C3H/HeN,1)	25 2-AG (18 µg), 25 Vehicle 7 AEA (1—10 µg), 7 Vehicle	− 32.09 [− 47.38, − 16.80] − 19.26 [− 32.64, − 5.88]	− 24.83 [− 34.89, − 14.76]	Mean Difference (IV, Fixed, 95% CI)	Chi^2^ = 1.53, df = 1 (*p* = 0.22); I^2^ = 34.7%	Z = 4.83 (*p* < 0.00001)
Synthetic cannabinoid receptor agonists	Paw withdrawal threshold (g)	Female (Rats)	CB2-selective agonist (Sprague–Dawley, 1) CB1-selective agonist (Sprague–Dawley, 1) CB1/2 agonist (Sprague–Dawley, 1)	25 AM1241 (0.06 nmol/l), 25 Vehicle 16 JWH015 (10 µg), 16 Vehicle 25 ACEA (8.2 nmol/l), 25 Vehicle 12 WIN 55,212-2 (30 µg), 12 Vehicle	8.94 [6.49, 11.39] 6.80 [5.00, 8.61] 6.74 [2.65, 10.83] 10.43 [9.29, 11.57]	8.18 [6.14, 10.21]	Mean Difference (IV, Random, 95% CI)	Chi^2^ = 12.66, df = 3 (*p* = 0.005), I^2^ = 76.3%	Z = 7.88 (*p* < 0.00001)
		Male (Mice)	CB2-selective agonist (C3H/HeJ, 4)	12 AM1241 (6 mg/kg/day, 3 days), 12 Vehicle 26 JWH015 (2 µg), 26 Vehicle	0.81 [0.30, 1.32] 0.89 [0.79, 1.00]	0.89 [0.79, 0.99]	Mean Difference (IV, Fixed, 95% CI)	Chi^2^ = 0.10, df = 1 (*p* = 0.75), I^2^ = 0%	Z = 16.90 (*p* < 0.00001)
	Paw withdrawal frequency (%)	Male (Mice)	CB2-selective agonist (C3H/HeNCr MTV-, 1) CB1-selective agonist (C3H/HeNCr MTV-, 1) CB1/2 agonist (C3H/HeNCr MTV-, 1) CB1/2 agonist (C3H/He, 1)	5 AM1241 (60 µg), 5 Vehicle 6 ACPA (60 µg), 6 Vehicle 6 CP55,940 (1 mg/kg), 6 Vehicle 7 WIN55,212-2 (10 µg), 7 Vehicle	− 24.93 [− 40.59, − 9.27] − 26.50 [− 32.53, − 20.47] − 52.38 [− 62.03, − 42.73] − 10.20 [− 21.44, 1.04]	− 28.73 [− 45.43, − 12.02]	Mean Difference (IV, Random, 95% CI)	Chi^2^ = 34.20, df = 3 (*p* < 0.00001), I^2^ = 91.2%	Z = 3.37 (*p* = 0.0008)
	Paw withdrawal thermal latency (s)	Male (Rats)	CB2 agonist (Wistar rats, 3)	30 AM1241 (7 µg), 30 Vehicle	NA	3.94 [2.13, 5.75]	Mean Difference (IV, Random, 95% CI)	Chi^2^ = 7.24, df = 2 (*p* = 0.03); I^2^ = 72%	Z = 4.27 (*p* < 0.0001)
	Number of spontaneous flinches (/2 min)	Male (Mice)	CB2 agonist (C3H/HeN, 3) CB2 agonist (C3H/HeJ, 1) CB1 agonist (C3H/HeN, 1)	12 AM1241 (6 mg/kg), 12 Vehicle 14 JWH015 (2 µg), 14 Vehicle 7 ACEA (1 nmol), 7 Vehicle	− 5.49 [− 7.13, − 3.85] − 3.07 [− 3.60, − 2.55] − 8.49 [− 10.13, − 6.85]	− 4.85 [− 6.74, − 2.96]	Mean Difference (IV, Random, 95% CI)	Chi^2^ = 42.74, df = 2 (*p* < 0.00001), I^2^ = 95.3%	Z = 5.03 (*p* < 0.00001)
	Score of ambulatory	Female (Rats)	CB2 agonist (Sprague–Dawley rats, 2)	16 JWH015 (10 µg), 16 Vehicle	NA	− 1.71 [− 3.07, − 0.36]	Mean Difference (IV, Random, 95% CI)	Chi^2^ = 16.67, df = 1 (*p* < 0.0001); I^2^ = 94%	Z = 2.49 (*p* = 0.01)

*MD* mean difference, *Std.* standardized, *IV* inverse-variance weighting, *NA* not applicable, *s* seconds, *g* gram.

**Table 3:** Articles included in narrative synthesis.

Study type/References		Title
Animal studies	de Almeida et al.^87^	Characterization of Cancer-Induced Nociception in a Murine Model of Breast Carcinoma
	Guerrero et al.^88^	Peripheral cannabinoids attenuate carcinoma-induced nociception in mice
	Hald et al.^84^	Differential effects of repeated low dose treatment with the cannabinoid agonist WIN 55,212-2 in experimental models of bone cancer pain and neuropathic pain
	Ji et al.^91^	Bufalin attenuates cancer-induced pain and bone destruction in a model of bone cancer
	Jiang et al.^92^	Morin Suppresses Astrocyte Activation and Regulates Cytokine Release in Bone Cancer Pain Rat Models
	Lu et al.^90^*	A Single Intrathecal or Intraperitoneal Injection of CB2 Receptor Agonist Attenuates Bone Cancer Pain and Induces a Time-Dependent Modification of GRK2
	Saghafiet al.^89^	Cannabinoids attenuate cancer pain and proliferation in a mouse model
	Uhelski et al.^85^	The non-selective cannabinoid receptor agonist WIN 55,212-2 attenuates responses of C-fiber nociceptors in a murine model of cancer pain
	Wang et al.^86^	Role of cannabinoid 2 receptor in the development of bone cancer pain
	Khasabova et al.^83^*	Increasing 2-arachidonoyl glycerol signaling in the periphery attenuates mechanical hyperalgesia in a model of bone cancer pain
Human studies	Anderson et al.^93^	Impact of Medical Cannabis on Patient-Reported Symptoms for patients with Cancer Enrolled in Minnesota’s Medical Cannabis Program
	Aviram et al.^94^	Short-Term Medical Cannabis Treatment Regimens Produced Beneficial Effects among Palliative Cancer Patients
	Bar-Lev Schleider et al.^95^	Prospective analysis of safety and efficacy of medical cannabis in large unselected population of patients with cancer
	Bar-Sela et al.^96^	The medical necessity for medicinal cannabis: prospective, observational study evaluating the treatment in cancer patients on supportive or palliative care
	Bar-Sela et al.^97^	The Effects of Dosage-Controlled Cannabis Capsules on Cancer-Related Cachexia and Anorexia Syndrome in Advanced Cancer Patients: Pilot Study
	Calcaterra et al.^98^	A population-based survey to assess the association between cannabis and quality of life among colorectal cancer survivors
	Chapman et al.^103^	Medical cannabis in pediatric oncology: a survey of patients and caregivers
	Côté et al.^104^	Improving Quality of Life With Nabilone During Radiotherapy Treatments for Head and Neck Cancers: A Randomized Double-Blind Placebo-Controlled Trial
	Davies et al.^99^	A Pilot Study of Orally Administered a1-trans-tetrahydrocannabinol in the Management of Patients Undergoing Radiotherapy for Carcinoma of the Bronchus
	Donovan et al.^113^	Cannabis Use in Young Adult Cancer Patients
	Donovan et al.^114^	Relationship of Cannabis Use to Patient-Reported Symptoms in Cancer Patients Seeking Supportive/Palliative Care
	Elliott et al.^100^	Medical marijuana use in head and neck squamous cell carcinoma patients treated with radiotherapy
	Good et al.^101^	An Open-Label Pilot Study Testing the Feasibility of Assessing Total Symptom Burden in Trials of Cannabinoid Medications in Palliative Care
	Grimison et al.^102^	Oral THC:CBD cannabis extract for refractory chemotherapy-induced nausea and vomiting: a randomised, placebo-controlled, phase II crossover trial
	Jochimsen et al.^115^	Effect of benzopyranoperidine, a Δ-9-THC congener, on pain
	Johnson et al.^116^	An open-label extension study to investigate the long-term safety and tolerability of THC/CBD oromucosal spray and oromucosal THC spray in patients with terminal cancer-related pain refractory to strong opioid analgesics
	Meghani et al.^117^	Impact of Cannabis Use on Least Pain Scores Among African American and White Patients with Cancer Pain: A Moderation Analysis
	Ofir et al.^118^	Medical marijuana use for pediatric oncology patients: single institution experience
	Pawasarat et al.^119^	The Efficacy of Medical Marijuana in the Treatment of Cancer-Related Pain
	Raghunathan et al.^120^	In the weeds: a retrospective study of patient interest in and experience with cannabis at a cancer center
	Tavhare et al.^121^	Management of chronic pain with water-wash processed *Cannabis sativa* L. in cancer patients with deprived quality of life: An open-label single arm clinical trial
	Turcott et al.^122^	The effect of nabilone on appetite, nutritional status, and quality of life in lung cancer patients: a randomized, double-blind clinical trial
	Waissengrin et al.^123^	Patterns of Use of Medical Cannabis Among Israeli Cancer Patients: A Single Institution Experience
	Webster et al.^124^	Prescribed medical cannabis in women with gynecologic malignancies: A single-institution survey-based study
	Wiseman et al.^125^	The Effect of Preoperative Cannabis Use on Postoperative Pain Following Gynaecologic Oncology Surgery

*Indicates articles reported studies that also included in the present meta-analysis.

### Quality assessment

Risk of bias for in vivo studies was assessed using the Syrcle risk of bias tool^75^ (Figure S1). Out of 10 items, 4 items scored as ‘unknown risk’ for over 50% in vivo studies. These were item 1—Sequence generation, item 3—Allocation concealment, item 5—Blinding (Performance bias), and item 6—Random outcome assessment. Although these quality items are imperative for high quality clinical studies, we believe that it is rather uncommon for in vivo studies to fulfill these; hence their prevalence amongst the included in vivo studies here did not surprise us. If we exclude the four items that most articles scored ‘unclear risk’ (items 1, 3, 5 and 6), all articles indicated an overall high quality and hence were not excluded based solely on their quality. Risk of bias for randomized controlled trials (RCTs) and crossover studies was assessed by the Cochrane risk-of-bias tool for randomized trials (RoB 2), whereas for quasi-experimental, cohort, retrospective and observational studies was assessed using a modified Newcastle–Ottawa Scale (NOS). The overall quality of the human studies was mostly moderate to high (for 29 out of 35 papers [~ 83%]). Only 6 papers (17%) were judged to be of low quality due to high risk of bias. Details of quality assessment can be seen in Tables S8–S9 and Figs. S1–S2.

### Certainty of evidence

The outcomes of animal studies that examined the effects of CB_1/2_ receptor modulation on cancer-induced pain were uncertain due to low study number and/or high heterogeneity of models described pain (low certainty of evidence). Similarly, the effects of administration of CB_1/2_ agonists in human were uncertain (very low certainty of evidence) due to the small number of enrolled participants and the non-randomized design for half of the clinical studies.

### Meta-analysis of outcomes

#### Bone pain in animal models of cancer

The present meta-analysis and systematic review identified 29 articles that examined the pharmacological effects of endocannabinoids (2 articles), synthetic agonist (22 articles) and antagonist/inverse agonists (5 articles) of the classical CB_1/2_ receptors on mechanical allodynia (25 articles), thermal hyperalgesia (9 articles) and spontaneous pain (6 articles) in adult males (23 articles) and females (6 articles) rodents (mice, 16 and rats, 13 articles) (Tables 1, 2 and S2). All animal studies reported experiments in rodents bearing osteolytic cancer cells (Mouse—Fibrosarcoma (15 articles) and 4T1 (1 article) cells; Rat—Walker 256 (10 articles) and mammary MRMT-1 (3 studies) gland carcinoma cells) (Tables 1, 2 and S2).

##### Analgesic effects of endocannabinoids

Pooled analysis of studies in models of mechanical allodynia showed that intrathecal injection of the endocannabinoids 2-AG (MD − 32.09, ^95%^CI − 47.38, − 16.80) and AEA (MD − 19.26, ^95%^CI − 32.64, − 5.88) (CB_1/2_-non-selective) is associated with significant reduction in paw withdrawal frequency in adult male C3H/HeN mice inoculated with osteolytic sarcoma cells. Pooled analysis of these studies confirms that administration of the aforementioned endocannabinoids is associated with anti-allodynic effects in male mice bearing osteolytic tumours (MD − 24.83, ^95%^CI − 34.89, − 14.76), with a Z value of 4.83, which corresponds to a *p* value of *p* < 0.00001 (Table 2). Furthermore, a review of studies that were considered heterogeneous to pool or not reported in a format suitable for pooling (Table 3) confirmed that administration of the monoacylglycerol lipase (MAGL) inhibitor JZL184, which is known to enhance the levels of 2-AG^83^, reduced paw withdrawal frequency in male mice inoculated with osteolytic sarcoma cells^84^ (Table 3). Collectively, these findings show that activation of CB_1/2_ receptors by the endocannabinoids AEA and 2-AG is associated with reduced cancer-induced bone pain in the rodent models described.

##### Analgesic effects of synthetic CB_1/2_ agonists

Pooled analysis of studies that examined the effects of synthetic agonists of CB_1/2_ in models of mechanical allodynia also showed that administration of the CB_1_-selective ACPA (MD − 26.50, ^95%^CI − 32.53, − 20.47), CB_2_-selective AM1241 (MD − 24.93, ^95%^CI − 40.59, − 9.27), and CB_1/2_ selective CP55,940 (MD − 52.38, ^95%^CI − 62.03, − 42.73) and WIN55,212-2 (MD − 10.20, ^95%^CI − 21.44, − 1.04) agonists was associated with reduced paw withdrawal frequency in male mice bearing osteolytic cancer (overall MD − 28.73, ^95%^CI − 45.43, − 12.02), with a Z value of 3.37, which corresponds to a *p* value of less than *p* = 0.0008 (Table 2). Similarly anti-allodynic effects was observed in female rats treated with the CB_2_-selective AM1241 (MD 8.94, ^95%^CI 6.49, 11.39) and JWH015 (MD 6.80, ^95%^CI 5.00, 8.61), and CB_1/2_-selective ACEA (MD 6.74, ^95%^CI 2.65, 10.83) and WIN55,212-2 (MD 10.43, ^95%^CI 9.29, 11.57) agonists and exhibited significant increase in paw withdrawal threshold (overall MD 8.18, ^95%^CI 6.14, 10.21), with a Z value of 7.88, which corresponds to a *p* value of less than 0.00001 (Table 2). Similar anti-allodynic effects was observed in male mice treated with AM1241 (MD 0.81, ^95%^CI 0.30, 1.32) or JWH015 (MD 0.89, ^95%^CI 0.79, 1.00) with an overall MD of 0.89, ^95%^CI 0.79, 0.99, and a Z value of 16.90, which corresponds to a *p* value of less than 0.00001 (Table 2). In models of thermal hyperalgesia, analysis of pooled studies showed that administration of the CB_2_-selective AM1241 (MD 3.94, ^95%^CI 2.13, 5.75) is associated with increased paw withdrawal thermal latency in male rats, with a Z value of 4.27, which corresponds to a *p* value of less than 0.0001 (Table 2). Similarly, administration of the CB_2_-selective AM1241 (MD − 5.49, ^95%^CI − 7.13, − 3.85) and JWH015 (MD − 3.07, ^95%^CI − 3.60, − 2.55) as well as the CB_1_-selective ACEA (MD − 8.49, ^95%^CI − 10.13, − 6.85) agonists in male mice was associated with reduced number of spontaneous flinches (overall MD − 4.85, ^95%^CI − 6.74, − 2.96), with a Z value of 5.03, which corresponds to a *p* value of less than 0.00001 (Table 2). Our analysis also showed that administration of the CB_2_-selective JWH015 (MD − 1.71, ^95%^CI − 3.07, − 0.36) is associated with decreased ambulatory score in female rats, with a Z value of 2.49, which corresponds to a *p* value of 0.01 (Table 2). Overall, our present investigation suggests that synthetic agonists of CB_1/2_ exhibit analgesic effects in the described cancer models of mechanical and thermal allodynia in rodents. Summary of meta-analysis showing non-significant association of CB_1/2_ modulation and cancer-induced pain in animal models is shown in Table S6. A review of studies that were considered to be too heterogeneous to be included in the meta-analysis or not reported in a format unsuitable for pooling (Table 3) complement this conclusion by showing that administration of the endocannabinoid 2-AG or increasing its level by treatment with the MAGL inhibitor JZL-184 significantly reduced cancer-induced bone pain in mice, whereas AEA had no effects^84^_._ Consistently, administration of the CB_1/2_-selective WIN55,212-2, ACEA, and AM1241 attenuated bone pain in mouse models of primary sarcoma^85–87^, and metastases of breast, oral and lungs cancer^88–90^. The CB_2_-selective agonist JWH-015 also reduced cancer-induced bone pain in rats, and this effect was inhibited by the CB_2_-selective inverse/agonist AM630, suggesting a CB_2_ mediated effect^91^. AM630 also reduced the analgesic effects of natural anti-inflammatory agents such as Bufalin^92^ and Morin^93^, which have been found to modulate CB_2_ receptors.

##### Effectiveness of cannabinoid ligands in rodents

To evaluate the effectiveness of cannabinoids assessed in the meta-analysis, we compared the (std) MD of different interventions:

When different interventions applied to male mice and paw withdraw frequency (%) was measured as an outcome of pain, the effectiveness order is: CP 55,940 (− 52.38 [− 62.03, − 42.73]) > 2-AG (− 32.09 [− 47.38, − 16.80]) > ACPA (− 26.5 [− 32.53, − 20.47]) > AM1241 (− 24.93 [− 40.59, − 9.27]) > AEA (− 19.26 [− 32.64, − 5.88]) > WIN 55,212-2 (− 10.2 [− 21.44, 1.04]).

When different interventions applied to female rats and paw withdraw threshold (g) was measured as an outcome of pain, the effectiveness order is: WIN 55,212-2 (10.43[9.29, 11.57]) > AM1241 (8.94[6.49, 11.39]) > JWH015 (6.8[5.00, 8.61]) > ACEA (6.74[2.65, 10.83]).

When different interventions applied to male mice and paw withdraw threshold (g) was measured as an outcome of pain, the effectiveness order is: JWH015 (0.89[0.79, 1.00]) > AM1241 (0.81[0.30, 1.32]) and the difference is very small.

When different interventions applied to male mice and number of spontaneous flinches (/2 min) was measured as an outcome of pain, the effectiveness order is: ACEA (− 8.49 [− 10.13, − 6.85]) > AM1241 (− 5.49 [− 7.13, − 3.85]) > JWH015 (− 3.07 [− 3.60, − 2.55]).

According to the orders above, the same treatment produced varying degrees of effectiveness as measured by different pain parameters. The pain relief degree when applying the same cannabinoid also varies in different species of animals. For example, AM1241 > ACEA in paw withdraw threshold (g) of female rats (point 1), whereas ACEA > AM1241 in number of spontaneous flinches (/2 min) of male mice (point 3). Thus, we can’t simply draw a conclusion of the effectiveness of cannabinoids based on current results.

#### Pain in cancer patients

##### Regulation of cancer-induced pain in human by CB_1/2_ modulators

The present systematic review identified 35 human studies that examined the effects of medical cannabis (15), delta-9-THC/CBD extracts (4), delta-9-THC extracts alone (4), Sativex (or Nabiximols) (3), Nabilone (Cesamet) (2), Benzopyranoperidine (1), NIB (nitrogen analogue of delta-9-THC), water-wash processed *Cannabis sativa* L. leaves powder (1), or regular cannabis use (5) on pain scores [Numeric Rating Scale (NRS) (8), Edmonton Symptom Assessment Scale (ESAS) (6), Visual analogue scale (VAS) (2), Pain symptom scale 0–100 (2), Assessment of Quality of Life-8 dimensions (AQoL-8D) pain analysis (1), FACES Pain Rating Scale for pain (1)], Pain reduction (2) and Pain intensity (2) in cancer patients (Table 1 and Fig. 2). Summary of meta-analysis of included studies showing non-significant association of cancer-related bone pain with treatment with pharmacological modulators of CB_1_ and/or CB_2_ receptors in humans is shown in Table S7.

##### Analgesic effects of delta-9 THC and its synthetic derivative in humans

Pooled analysis of clinical studies in cancer patients (n = 44 in total) confirmed that treatment with delta-9-THC at a dose of 20 mg is associated with significant reduction in cancer-induced pain intensity compared to placebo, with a mean difference of 3.29 (^95%^CI 2.24, 4.33), and Z value of 6.17, which corresponds to a *p* value less than 0.00001 (Fig. 2, top panel). Our meta-analysis of pooled analysis also showed that treatment of cancer patients (n = 44 in total) with the nitrogen-containing benzopyran derivative, a modification of delta-1-trans-THC (NIB) at a dose of 4 mg is associated with significant reduction in cancer-induced pain intensity compared to placebo, with a mean difference of 2.55 (^95%^CI 1.58, 3.51), and Z value of 5.17, which corresponds to a *p* value less than 0.00001 (Fig. 2, middle panel).

##### Analgesic effects of medical cannabis in humans

Four clinical studies (1 RCT, 2 prospective and 1 retrospective cohort studies) investigated whether treatment with medical cannabis impact cancer-induced pain in female and male cancer patients (Fig. 2, bottom panel). Analysis of pooled studies from 277 cancer patients confirmed that treatment with medical cannabis is associated with significant reduction in cancer-induced pain compared to baseline after 3 months, with a standardized mean difference of − 0.19 (^95%^CI − 0.35, − 0.02), and Z value of 2.20, which corresponds to a *p* value of 0.03 (Fig. 2, bottom panel). A review of non-pooled prospective, retrospective, observational cross-sectional and pilot studies, together with questionnaires and surveys (Table 3) indicates that consumption of plant cannabis is well-tolerated for up to 2 years, and show that smoking, vaporizing, and consumption of medical capsules or homemade concentrated oil of cannabis or plant-derived delta-9-THC- and/or CBD-dominant products is associated with reduction of cancer-related pain in adults^94–103^, and children^104^. In stark contrast to these studies, other investigators observed that the synthetic cannabinoid nabilone failed to affect pain severity in cancer patients^105^. Altogether, findings from included and non-pooled studies provide pharmacological evidence that consumption of medical preparations containing natural cannabis, delta-9-THC and/or CBD is of value in the management of malignancy related symptoms, including pain.

### Bioinformatics validation

**Figure 3:**
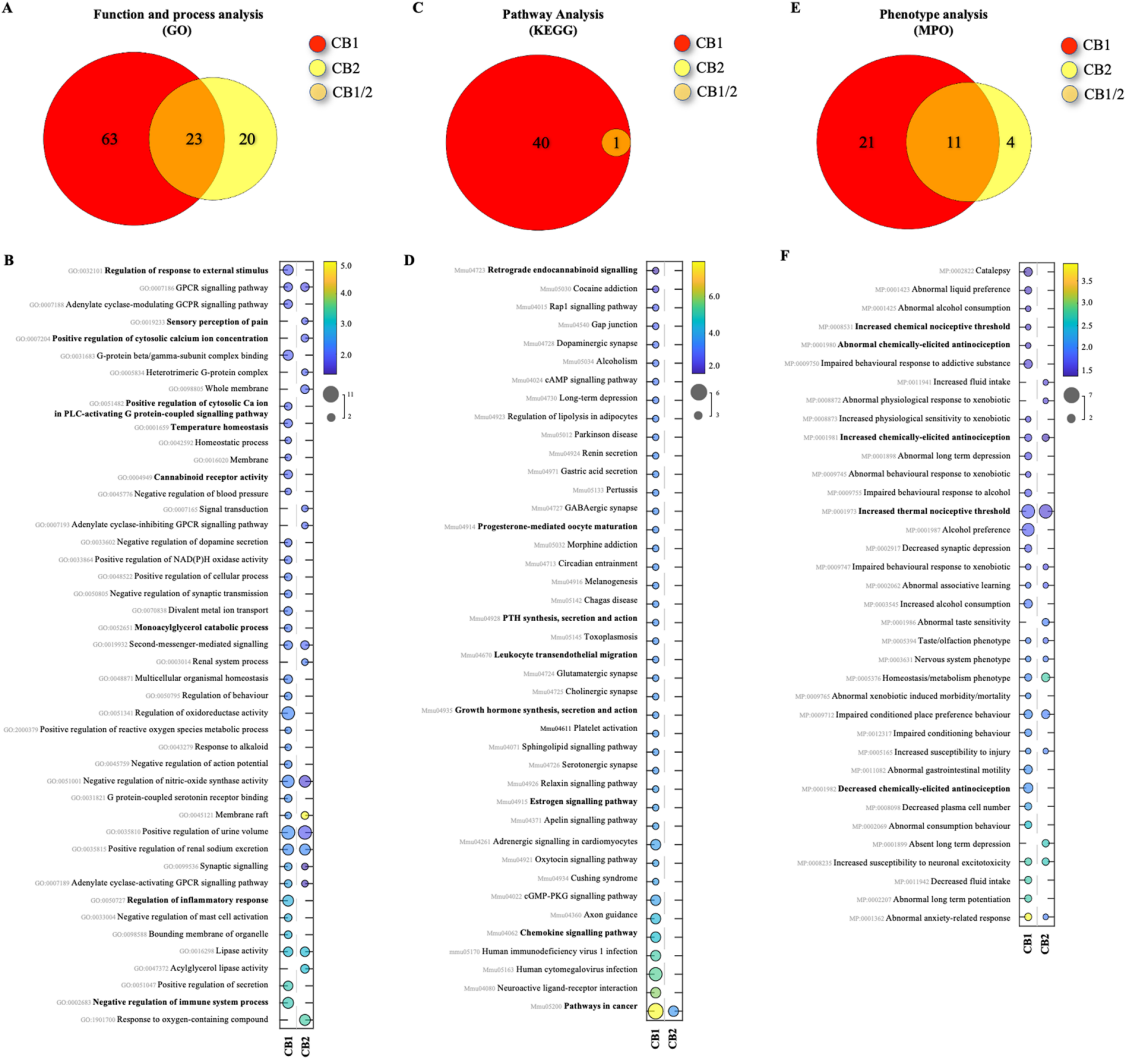
KEGG, GO and MPO enrichment analyses in mice. (**A**,**C**,**E**) Venn diagrams of enriched pathways or processes number of predicted associated genes of CNR1/2 (PAGs). PAGs are represented by different colours, and the numbers in the circles (and overlapping area) represent the number of uniquely enriched or shared pathways or processes. (**B**,**D**,**F**) Bubble charts of significantly enriched pathways, processes, functions, and diseases. The vertical axis represents the KEGG (**B**), GO (**D**) and MPO (**F**) pathway classification, and abscissa represents enrichment score, size of spots indicates the number of matchings, and circle colour represents the value of − log10 (False Discovery Rate, FDR). Only the top 45 gene functions with the highest enrichment were drawn in each plot. Permission has been obtained for using KEGG pathway database (Kanehisa Laboratories, www.kegg.jp/kegg/kegg1.html).

**Figure 4:**
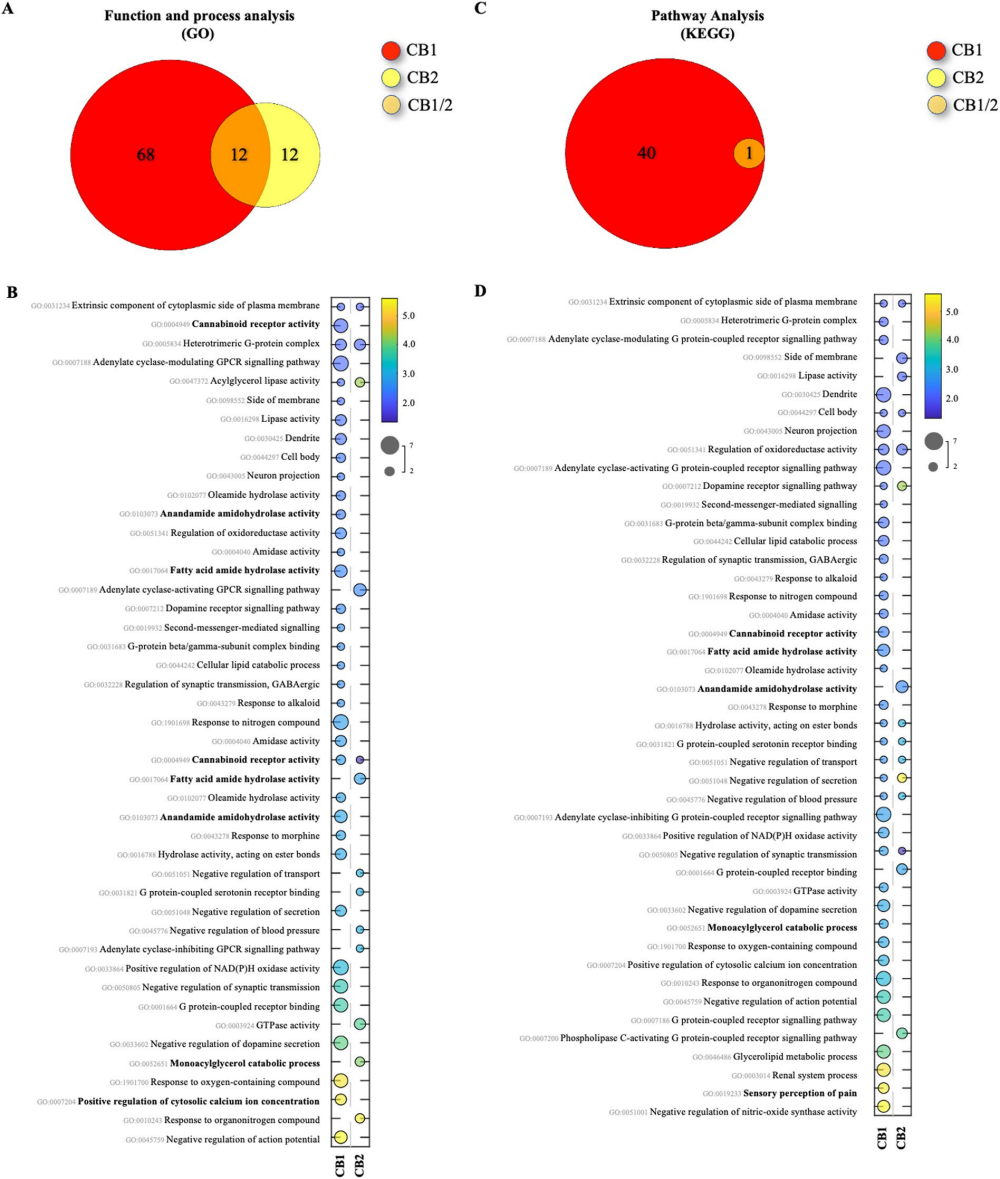
KEGG, GO and MPO enrichment analyses in rats. (**A**,**C**,**E**) Venn diagrams of shared pathways or processes. (**B**,**D**,**F**) Bubble charts of significantly enriched pathways, processes, and functions. For more information refer to legend for Fig. 3.

**Figure 5:**
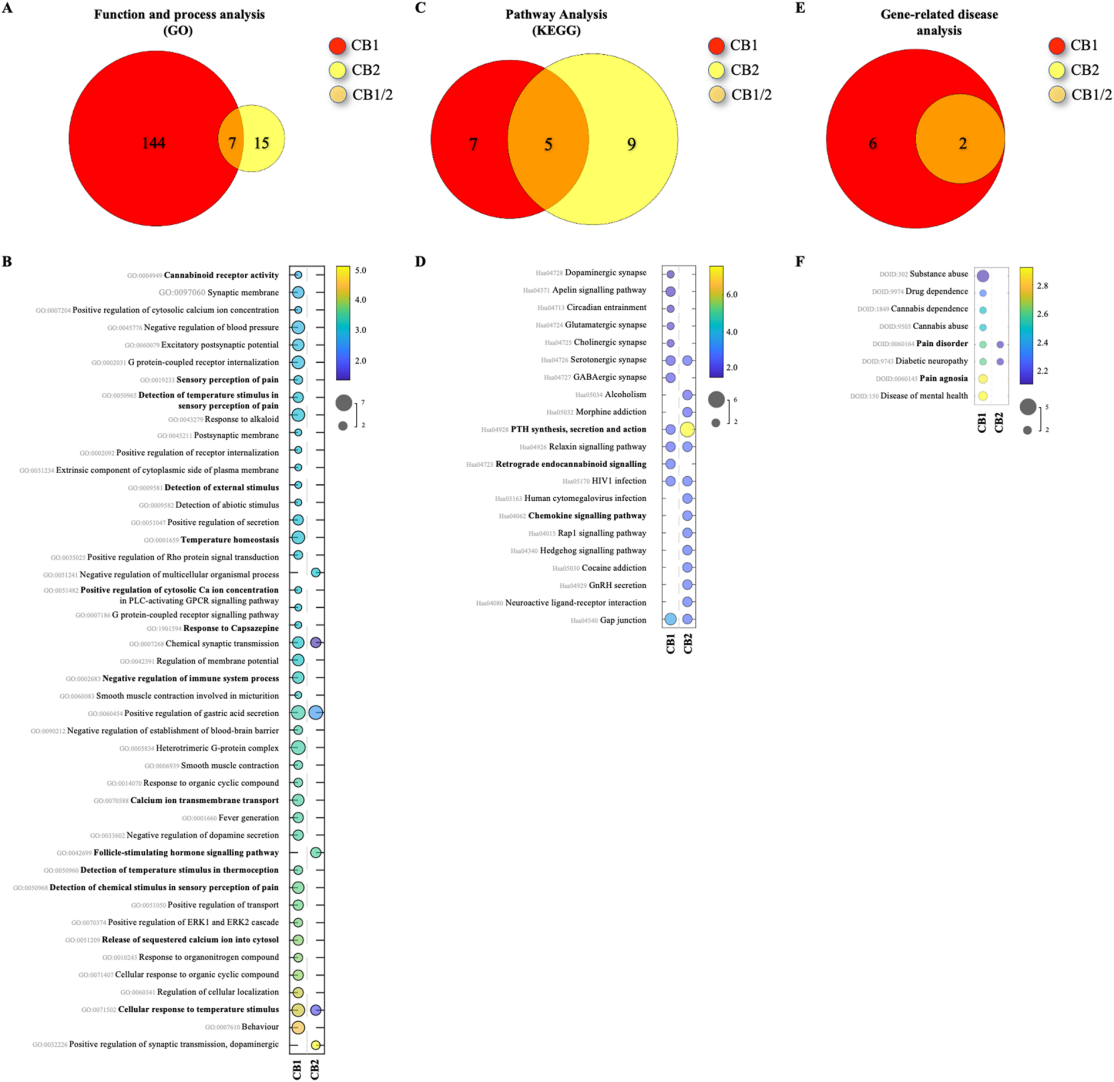
KEGG, GO and MPO enrichment analyses in human. (**A**,**C**,**E**) Venn diagrams of shared pathways or processes. CB1 and CB2 receptors are represented by different colours, and the numbers in the circles (and overlapping area) represent the number of uniquely expressed or shared genes. (**B**,**D**,**F**) Bubble charts of significantly enriched pathways, processes, functions, and diseases. For more information refer to legend for Fig. 3.

Next, we conducted a bioinformatics analysis of KEGG, GO, MPO, and gene-related diseases databases to gain an insight of the potential signalling transduction pathways, functions, processes, and diseases affected by modulation of the classical CB_1/2_ receptors. A total of 364 functions and processes in GO (CB_1_: 317 and CB_2_: 89), 101 pathways in KEGG (CB_1_: 92 and CB_2_: 16), 36 gene phenotypes in MPO (CB_1_: 32 and CB_2_: 15), and 8 gene-related diseases in humans (CB_1_: 8 and CB_2_: 2) databases were obtained (Figs. 3, 4 and 5, panels A, C, E).

### GO analysis for CB_1/2_ receptors in rodents and human

The Gene Ontology (GO) enrichment analysis revealed that the classical CB_1_ and CB_2_ receptors were significantly enriched in a total of 106 (CB_1_: 86 and CB_2_: 43), 92 (CB_1_: 80 and CB_2_: 24) and 166 (CB_1_: 151 and CB_2_: 22) functions and processes in mouse, rat, and human datasets, respectively (Figs. 3, 4 and 5, panels A). Further examination of terms among the three species showed that CB_1_ and CB_2_ enrichment is associated with sensory perception of pain (CB_1/2_), temperature (CB_1_/CB_2_) and external stimulus (CB_1_), as well as other processes implicated in nociception such as synaptic transmission (CB_1/2_), calcium homeostasis (CB_1/2_), and response to modulators of the transient receptor potential cation (TRPV) channels, namely Capsazepine (CB_1_) (Figs. 3, 4 and 5, panels B). Our analysis also confirmed that the enrichment of CB_1/2_ receptors in (endo)cannabinoid signalling (CB_1/2_), synthesis (AEA amidohydrolase activity, CB_1_) and metabolism (Fatty acid amide hydrolase activity, CB_1_ and Monoacylglycerol catabolic process, CB_1/2_), together with a complex network of endocrine (Follicle-stimulating hormone (FSH) activity, CB_2_), immuno-modulatory (CB_1_), inflammatory (CB_1_), and G-protein-coupled receptors (GPCR)-related signalling processes (Figs. 3, 4 and 5, panels B).

### KEGG analysis for CB_1/2_ receptors in rodents and humans

Next, we utilized KEGG enrichment analysis to reveal that CB_1_ and CB_2_ receptors were significantly enriched in a total of 40 (CB_1_: 40 and CB_2_: 1), 40 (CB_1_: 40 and CB_2_: 1) and 21** (**CB_1_: 12 and CB_2_: 14**)** signalling pathways were significantly enriched in mouse, rat and human, respectively (Figs. 3, 4 and 5, panels C), and to confirm the association of these receptors with a wide range of signal transduction pathways (Figs. 3, 4 and 5, panels D) that includes cancer-specific (CB_1/2_), retrograde endocannabinoid (CB_1_), immuno-modulatory (CB_1/2_), and GPCR-related (CB_1/2_) signalling pathways (Figs. 3, 4 and 5, panels D). Notably, the list also includes signalling pathways for growth hormones (CB_1_), parathyroid hormone (PTH, CB_1/2_), oestrogen, relaxin, oxytocin, and progesterone (CB_1_), which have been found to regulate bone remodelling^106–108^.

### MPO analysis for CB_1/2_ receptors in rodents

We also carried out Monarch Phenotype Ontology (MPO) analysis in rodents to show that CB_1_ and CB_2_ receptors were significantly enriched in a total of 36 (CB_1_: 32 and CB_2_: 15) phenotypes in mouse (Fig. 3, panel E). Close examination of the terms confirmed that the enrichment of CB_1_ and CB_2_ is associated with chemical (CB_1/2_) and thermal (CB_1/2_) induced nociception (Fig. 3, panel F).

### Gene-related disease analysis for CB_1/2_ receptors in humans

Finally, analysis of gene-related diseases in humans revealed the enrichment of CB_1_ and CB_2_ receptors in a total of 8 (CB_1_: 8 and CB_2_: 2) diseases that include pain agnosia (CB_1_) and disorder (CB_1/2_) (Fig. 5, panel F).

## Discussion

The collective meta-analysis and bioinformatics validation yielded 4 species-specific outcomes: (1) In mice, the endocannabinoids AEA and 2-AG (CB_1/2_ non-selective) and synthetic CB_1_-selective ACPA and ACEA, CB_2_-selective JWH015 and AM1241, and CB_1/2_ non-selective CP55,940 and WIN55,212-2 exhibited anti-allodynic effects in spontaneous and mechanical models of osteolysis-induced pain; (2) In rats, treatment with synthetic CB_1_-selective ACEA, CB_2_-selective JWH015 and AM1241, and CB_1/2_ non-selective WIN55,212-2 is associated with anti-allodynic effects in spontaneous, mechanical, and thermal models of osteolysis-induced pain; (3) In human, treatment of patients with delta-9-THC (20 mg), its nitrogen-containing derivative NIB (4 mg) or non-specified dose of medical cannabis is associated with significant reduction in cancer-induced intensity of pain, but not bone pain. (4) In the 3 species, the classical CB_1/2_ receptors—alone and in combination with other receptors and channels (Supplementary Fig. S3)—are implicated in the regulation of a complex network of pain related disorders, functions, processes, pathways, and phenotypes coupled with a set of sensory perception, cancer-specific, pro-inflammatory, and immuno-modulatory activities. Thus, we cautiously conclude that CB_1/2_ ligands attenuate cancer-induced bone pain in rodents, and pain intensity in cancer patients.

### Therapeutic implications

The classical CB_1/2_ receptors are the most studied members of the endocannabinoid system, and thus have been involved in the regulation of cancer-induced pain by a plethora of natural and synthetic cannabinoids^16,36,44–57^. Broadly, the results from the present study complement findings from previous studies and provide further support to the hypothesis that natural endocannabinoids, medical cannabis (marijuana), and synthetic ligands of CB_1/2_ alleviate pain in cancer patients and rodents bearing tumours. Whilst it is tempting to conclude that CB_1/2_ activation is of benefit in the treatment of cancer-induced pain, we propose the followings: First, the analgesic effects of the CB_1/2_-non-selective endocannabinoids and synthetic CP55,940 and WIN55,212-2 should be validated in genetically modified animal models lacking CB_1_, CB_2_ and most importantly both receptors. Secondly, we recommend that the aforementioned studies to be conducted in conditional knockout animals that lack one or both CB_1/2_ receptors in tumour and/or peripheral cells such as immune, bone, and sensory nerve cells. Such approach will shed light on the cells and activities involved, gauge the ability of test agents to cause CB1-related adverse effects, hopefully provide support and guide the enthusiasm into the development of peripherally acting analogues or congeners of the agents featured in pooled studies.

Since the discovery of the link between CB_1_ receptor and osteoclastic bone resorption in 2005^40^, numerous studies have reported that osteoclasts, osteoblasts, immune, tumour and peripheral neurons in the skeleton express CB_1_ and CB_2_ receptors, and both genetic and pharmacological modulation of these receptors exerted paradoxical effects on bone cell activity and skeletal remodelling^32–39^. Thus, it is important that future studies should explore further the multi-modal capabilities of the CB_1/2_ agonists in the aforementioned experiments by attempting to establish if the analgesic effect of CB_1/2_-selective agents is due to or independent of anti-resorptive or osteoanabolic effects. An important finding of our study is that medical cannabis, delta-9-THC and NIB reduced pain intensity in cancer patients. This finding has a number of implications. First, it broadly supports the study hypothesis, but failed to provide further support to findings from the rodent studies included in the meta-analysis. It was surprising that only 2 human studies reported incidence of bone pain in cancer patients, and none of the terms uncovered by the bioinformatics analysis has a direct link to bone remodelling. Thus, the association between medical cannabis, CB_1/2_ receptors and bone pain in cancer patients remains unclear. For that, further studies are warranted. Finally, the bioinformatics analysis identified a wide range of central nervous system (CNS) and peripheral factors, pathways and processes that are likely to be involved in the actions of CB_1/2_ ligands included in the present investigation^15,19,109,110^. For example, chemokines, arachidonic acid metabolites, platelet activating factors, and the variety of circulating GPCR-activating ligands such as 2-AG, growth hormones, PTH, FSH, oestrogen, relaxin, and progesterone are involved in regulation of cancer-bone-immune-sensory nerve cell crosstalk^111^. We also uncovered a number of processes and proteins implicated in cell-to-cell adhesion and interactions, namely gap junction, regulation of transport, membrane embedded or anchored proteins and organelles, and signalling pathways such as Rap1 and Rho^112–114^. These findings are important because cancer-induced pain is a multi-factorial disorder, and bone pain is predominately caused by the ability of tumour cells and their derived factors to influence a diverse set of host cells and processes in the skeleton. Notwithstanding, further research must explore to what extent the multi-modal, multi-factorial, multi-cellular action of CB_1/2_ agonists may exert their analgesic effect directly and/or indirectly by regulating the activity of other neuronal or peripheral targets particularly the novel cannabinoid receptor GPR55 (aka CB_3_), and Transient receptor potential vanilloid 1 (TRPV1), which have been demonstrated to play an important role in the differentiation and death of bone and tumour cells^41,71,115–124^.

### Strengths and limitations

The strength of the present investigation is warranted by combining meta-analysis and bioinformatics validation to systematically and comprehensively examine the evidence from studies from 7 databases, namely Medline, Web of Science, Scopus, KEGG, GO, MPO and gene-related disorders. In meta-analysis, we used the online tool WebPlotDigitizer (https://apps.automeris.io/wpd/) to ensure that the mean and standard deviation (or standard error) measurement from all relevant figures in included studies were obtained. Thus, no data was deemed non-retrievable. We also took the decision to combine evidence from animal and human studies to explore the evidence from cancer studies that examined the effects of CB_1/2_ modulation on a wide range of pain indices using a variety of approaches, namely administration of pharmacological agents, genetic knockout of gene in animals, and manipulation of gene, Ribonucleic acid (RNA) and protein expression in patients. This lent more scrutiny to the proposed hypothesis and added credibility to outcomes. Notwithstanding, the present study has several limitations: (1) our search was restricted to articles written in English language; (2) the number of relevant human studies that described bone pain is low; (3) included animal studies only used xenograft models of local osteolysis and a limited number of strains; (4) different doses, route of administration, and treatment regimens were used in included animals and human studies; (5) low study number (< 10) was insufficient to perform meta-regression, Egger’s test or Funnel plot analysis. Thus, the evidence to conclusively support or refute the proposed hypothesis is insufficient.

## Conclusion

The use of cannabis among cancer survivors is on the rise. The *Cannabis sativa* plant however contains approximately 66 biologically-active substances including the CB_1/2_-non-selective delta-9-THC and CBD^111,125^. Thus, there is an urgent need to examine the efficacy, selectivity, and mechanisms by which both natural and synthetic cannabinoids exert their analgesic effects in animal models, and validate the analgesic potential of synthetic CB_1/2_-selective agents in cancer patients. Findings from the rodent studies included in the present meta-analysis confirmed that pharmacological—but not genetic—modulation of the classical CB_1_, CB_2_ receptors or both is associated with significant reduction in cancer-induced bone pain. However, we caution that low study number coupled with homogeneity of the animal experiments on which this conclusion is based, limit the translation of our findings into clinical practice. We, therefore, recommend that future studies should further validate the analgesic, as well as off-target, effects of the cannabinoid ligands featured in these studies in rodents lacking CB_1_, CB_2_, and both. In human, findings from included studies suggest that medical cannabis and the CB_1/2_-non-selective delta-9-THC and its synthetic derivative NIB reduce pain intensity in cancer patients. Whilst these results are encouraging, it is disappointing to discover that very few human studies evaluated bone pain. Thus, the association between selective modulation of CB_1_ or CB_2_ and bone pain in cancer patients remains unexplored. Nevertheless, our findings altogether indicate that CB_1/2_ ligands attenuate cancer-induced bone pain in rodents, and pain intensity in humans. These findings are confounded by lack of evidence from bone pain studies in metastatic cancers in patients and genetically engineered animals.

### Supplementary Information

Supplementary Information.

## Data Availability

The datasets used and analysed in the present study are available from the public sources described.

## References

[1] Nersesyan H, Slavin KV (2007). Current aproach to cancer pain management: Availability and implications of different treatment options. Ther. Clin. Risk Manag..

[2] Caraceni A (2001). Evaluation and assessment of cancer pain and cancer pain treatment. Acta Anaesthesiol. Scand..

[3] Caraceni A, Shkodra M (2019). Cancer pain assessment and classification. Cancers (Basel).

[4] Dy SM (2008). Evidence-based standards for cancer pain management. J. Clin. Oncol..

[5] Burton AW, Fanciullo GJ, Beasley RD, Fisch MJ (2007). Chronic pain in the cancer survivor: A new frontier. Pain Med..

[6] Lozano-Ondoua AN, Symons-Liguori AM, Vanderah TW (2013). Cancer-induced bone pain: Mechanisms and models. Neurosci. Lett..

[7] Mantyh P (2013). Bone cancer pain: Causes, consequences, and therapeutic opportunities. Pain.

[8] Davila D, Antoniou A, Chaudhry MA (2015). Evaluation of osseous metastasis in bone scintigraphy. Semin. Nucl. Med..

[9] Li BT, Wong MH, Pavlakis N (2014). Treatment and prevention of bone metastases from breast cancer: A comprehensive review of evidence for clinical practice. J. Clin. Med..

[10] Mercadante S (1997). Malignant bone pain: Pathophysiology and treatment. Pain.

[11] Sterling JA, Edwards JR, Martin TJ, Mundy GR (2011). Advances in the biology of bone metastasis: How the skeleton affects tumor behavior. Bone.

[12] Zajaczkowska R, Kocot-Kepska M, Leppert W, Wordliczek J (2019). Bone pain in cancer patients: Mechanisms and current treatment. Int. J. Mol. Sci..

[13] Aielli F, Ponzetti M, Rucci N (2019). Bone metastasis pain, from the bench to the bedside. Int. J. Mol. Sci..

[14] Clohisy DR, Mantyh PW (2003). Bone cancer pain. Clin. Orthop. Relat. Res..

[15] Oostinga D, Steverink JG, van Wijck AJM, Verlaan JJ (2020). An understanding of bone pain: A narrative review. Bone.

[16] Ellingson HM, Vanderah TW (2020). Potential therapeutic treatments of cancer-induced bone pain. Curr. Opin. Support Palliat. Care.

[17] Figura N, Smith J, Yu HM (2018). Mechanisms of, and adjuvants for, bone pain. Hematol. Oncol. Clin. N. Am..

[18] Gardner K, Laird BJA, Fallon MT, Sande TA (2019). A systematic review examining clinical markers and biomarkers of analgesic response to radiotherapy for cancer-induced bone pain. Crit. Rev. Oncol. Hematol..

[19] Yoneda T, Hiasa M, Nagata Y, Okui T, White FA (2015). Acidic microenvironment and bone pain in cancer-colonized bone. Bonekey Rep..

[20] Milgrom DP, Lad NL, Koniaris LG, Zimmers TA (2017). Bone pain and muscle weakness in cancer patients. Curr. Osteoporos. Rep..

[21] Rome S, Noonan K, Bertolotti P, Tariman JD, Miceli T (2017). Bone health, pain, and mobility: Evidence-based recommendations for patients with multiple myeloma. Clin. J. Oncol. Nurs..

[22] Pertwee RG (2006). Cannabinoid pharmacology: The first 66 years. Br. J. Pharmacol..

[23] Marx J (2006). Drug development. Drugs inspired by a drug. Science.

[24] Di Marzo V, Bifulco M, De Petrocellis L (2004). The endocannabinoid system and its therapeutic exploitation. Nat. Rev. Drug Discov..

[25] Di Marzo V (2008). Targeting the endocannabinoid system: To enhance or reduce?. Nat. Rev. Drug Discov..

[26] Pacher P, Batkai S, Kunos G (2006). The endocannabinoid system as an emerging target of pharmacotherapy. Pharmacol. Rev..

[27] Pagotto U, Marsicano G, Cota D, Lutz B, Pasquali R (2006). The emerging role of the endocannabinoid system in endocrine regulation and energy balance. Endocr. Rev..

[28] Cunha P, Romao AM, Mascarenhas-Melo F, Teixeira HM, Reis F (2011). Endocannabinoid system in cardiovascular disorders—New pharmacotherapeutic opportunities. J. Pharm. Bioallied Sci..

[29] Katchan V, David P, Shoenfeld Y (2016). Cannabinoids and autoimmune diseases: A systematic review. Autoimmun. Rev..

[30] Manzanares J, Julian M, Carrascosa A (2006). Role of the cannabinoid system in pain control and therapeutic implications for the management of acute and chronic pain episodes. Curr. Neuropharmacol..

[31] Rahn EJ, Hohmann AG (2009). Cannabinoids as pharmacotherapies for neuropathic pain: From the bench to the bedside. Neurotherapeutics.

[32] Atwood BK, Mackie K (2010). CB2: A cannabinoid receptor with an identity crisis. Br. J. Pharmacol..

[33] Bab I, Ofek O, Tam J, Rehnelt J, Zimmer A (2008). Endocannabinoids and the regulation of bone metabolism. J. Neuroendocrinol..

[34] Idris AI, Ralston SH (2010). Cannabinoids and bone: Friend or foe?. Calcif. Tissue Int..

[35] Marino S, Idris AI (2017). Emerging therapeutic targets in cancer induced bone disease: A focus on the peripheral type 2 cannabinoid receptor. Pharmacol. Res..

[36] Rossi F (2019). The endocannabinoid/endovanilloid system in bone: From osteoporosis to osteosarcoma. Int. J. Mol. Sci..

[37] Marino S (2020). JZL184, a monoacylglycerol lipase inhibitor, induces bone loss in a multiple myeloma model of immunocompetent mice. Calcif. Tissue Int..

[38] Marino S (2019). Paradoxical effects of JZL184, an inhibitor of monoacylglycerol lipase, on bone remodelling in healthy and cancer-bearing mice. EBioMedicine.

[39] Sophocleous A (2015). Bone cell-autonomous contribution of type 2 cannabinoid receptor to breast cancer induced osteolysis. J. Biol. Chem..

[40] Idris AI (2005). Regulation of bone mass, bone loss and osteoclast activity by cannabinoid receptors. Nat. Med..

[41] Rossi F (2009). The endovanilloid/endocannabinoid system in human osteoclasts: Possible involvement in bone formation and resorption. Bone.

[42] Idris AI (2009). Cannabinoid receptor type 1 protects against age-related osteoporosis by regulating osteoblast and adipocyte differentiation in marrow stromal cells. Cell Metab..

[43] Sophocleous A, Marino S, Kabir D, Ralston SH, Idris AI (2017). Combined deficiency of the Cnr1 and Cnr2 receptors protects against age-related bone loss by osteoclast inhibition. Aging Cell.

[44] Dumbili EW (2020). Cannabis normalization among young adults in a Nigerian city. J. Drug Issues.

[45] Hammond CJ, Chaney A, Hendrickson B, Sharma P (2020). Cannabis use among U.S. adolescents in the era of marijuana legalization: A review of changing use patterns, comorbidity, and health correlates. Int. Rev. Psychiatry.

[46] Klein A, Potter GR (2018). The three betrayals of the medical cannabis growing activist: From multiple victimhood to reconstruction, redemption and activism. Int. J. Drug Policy.

[47] Johnstad PG (2020). Cannabis as entheogen: Survey and interview data on the spiritual use of cannabis. J. Cannabis Res..

[48] Scourfield A (2019). Synthetic cannabinoid availability on darknet drug markets—Changes during 2016–2017. Toxicol. Commun..

[49] Palace ZJ, Reingold DA (2019). Medical cannabis in the skilled nursing facility: A novel approach to improving symptom management and quality of life. J. Am. Med. Dir. Assoc..

[50] Lynch ME, Cesar-Rittenberg P, Hohmann AG (2014). A double-blind, placebo-controlled, crossover pilot trial with extension using an oral mucosal cannabinoid extract for treatment of chemotherapy-induced neuropathic pain. J. Pain Symptom Manag..

[51] Sexton M, Garcia JM, Jatoi A, Clark CS, Wallace MS (2021). The management of cancer symptoms and treatment-induced side effects with cannabis or cannabinoids. J. Natl. Cancer Inst. Monogr..

[52] Do EK, Ksinan AJ, Kim SJ, Del Fabbro EG, Fuemmeler BF (2021). Cannabis use among cancer survivors in the United States: Analysis of a nationally representative sample. Cancer.

[53] Croker JA, Bobitt J, Arora K, Kaskie B (2022). Medical cannabis and utilization of nonhospice palliative care services: Complements and alternatives at end of life. Innov. Aging.

[54] Lichtman AH (2018). Results of a double-blind, randomized, placebo-controlled study of nabiximols oromucosal spray as an adjunctive therapy in advanced cancer patients with chronic uncontrolled pain. J. Pain Symptom Manag..

[55] Fallon MT (2017). Sativex oromucosal spray as adjunctive therapy in advanced cancer patients with chronic pain unalleviated by optimized opioid therapy: Two double-blind, randomized, placebo-controlled phase 3 studies. Br. J. Pain.

[56] Thompson AL (2020). The endocannabinoid system alleviates pain in a murine model of cancer-induced bone pain. J. Pharmacol. Exp. Ther..

[57] Sun J (2019). The endocannabinoid system: Novel targets for treating cancer induced bone pain. Biomed. Pharmacother..

[58] Matias I (2002). Presence and regulation of the endocannabinoid system in human dendritic cells. Eur. J. Biochem..

[59] Mechoulam R (2005). Plant cannabinoids: A neglected pharmacological treasure trove. Br. J. Pharmacol..

[60] Pertwee RG, Ross RA (2002). Cannabinoid receptors and their ligands. Prostaglandins Leukot. Essent. Fatty Acids.

[61] Mantyh PW (2019). Mechanisms that drive bone pain across the lifespan. Br. J. Clin. Pharmacol..

[62] Brazill JM, Beeve AT, Craft CS, Ivanusic JJ, Scheller EL (2019). Nerves in bone: Evolving concepts in pain and anabolism. J. Bone Miner. Res..

[63] Zheng XQ, Wu YH, Huang JF, Wu AM (2022). Neurophysiological mechanisms of cancer-induced bone pain. J. Adv. Res..

[64] Karanian DA, Brown QB, Makriyannis A, Bahr BA (2005). Blocking cannabinoid activation of FAK and ERK1/2 compromises synaptic integrity in hippocampus. Eur. J. Pharmacol..

[65] Derkinderen P (1996). Regulation of a neuronal form of focal adhesion kinase by anandamide. Science.

[66] Demuth DG, Molleman A (2006). Cannabinoid signalling. Life Sci..

[67] Gómez del Pulgar T, Velasco G, Guzmán M (2000). The CB1 cannabinoid receptor is coupled to the activation of protein kinase B/Akt. Biochem. J..

[68] Guzmán M, Galve-Roperh I, Sánchez C (2001). Ceramide: A new second messenger of cannabinoid action. Trends Pharmacol. Sci..

[69] Kapur A (2009). Atypical responsiveness of the orphan receptor GPR55 to cannabinoid ligands. J. Biol. Chem..

[70] Lauckner JE (2008). GPR55 is a cannabinoid receptor that increases intracellular calcium and inhibits M current. Proc. Natl. Acad. Sci. U.S.A..

[71] Ryberg E (2007). The orphan receptor GPR55 is a novel cannabinoid receptor. Br. J. Pharmacol..

[72] Moher D, Liberati A, Tetzlaff J, Altman DG, Group P (2009). Preferred reporting items for systematic reviews and meta-analyses: The PRISMA statement. Ann. Intern. Med..

[73] Deuis JR, Dvorakova LS, Vetter I (2017). Methods used to evaluate pain behaviors in rodents. Front. Mol. Neurosci..

[74] Staquet M, Gantt C, Machin D (1978). Effect of a nitrogen analog of tetrahydrocannabinol on cancer pain. Clin. Pharmacol. Ther..

[75] Hooijmans CR (2014). SYRCLE's risk of bias tool for animal studies. BMC Med. Res. Methodol..

[76] Wells G, Shea B, O’Connell D, Peterson J, Welch V, Losos M (2014). The Newcastle–Ottawa Scale (NOS) for assessing the quality of nonrandomised studies in meta-analyses. Appl. Eng. Agric..

[77] Ryan RHS (2016). How to GRADE the Quality of the Evidence.

[78] Hooijmans CR (2018). Facilitating healthcare decisions by assessing the certainty in the evidence from preclinical animal studies. PLoS ONE.

[79] Cumpston M (2019). Updated guidance for trusted systematic reviews: A new edition of the cochrane handbook for systematic reviews of interventions. Cochrane Database Syst. Rev..

[80] Szklarczyk D (2019). STRING v11: Protein–protein association networks with increased coverage, supporting functional discovery in genome-wide experimental datasets. Nucleic Acids Res..

[81] Shefchek KA (2020). The Monarch Initiative in 2019: An integrative data and analytic platform connecting phenotypes to genotypes across species. Nucleic Acids Res..

[82] Ucaryilmaz Metin C, Ozcan G (2022). Comprehensive bioinformatic analysis reveals a cancer-associated fibroblast gene signature as a poor prognostic factor and potential therapeutic target in gastric cancer. BMC Cancer.

[83] Mulvihill MM, Nomura DK (2013). Therapeutic potential of monoacylglycerol lipase inhibitors. Life Sci..

[84] Khasabova IA, Chandiramani A, Harding-Rose C, Simone DA, Seybold VS (2011). Increasing 2-arachidonoyl glycerol signaling in the periphery attenuates mechanical hyperalgesia in a model of bone cancer pain. Pharmacol. Res..

[85] Hald A (2008). Differential effects of repeated low dose treatment with the cannabinoid agonist WIN 55,212–2 in experimental models of bone cancer pain and neuropathic pain. Pharmacol. Biochem. Behav..

[86] Uhelski ML, Cain DM, Harding-Rose C, Simone DA (2013). The non-selective cannabinoid receptor agonist WIN 55,212–2 attenuates responses of C-fiber nociceptors in a murine model of cancer pain. Neuroscience.

[87] Wang D (2012). Role of cannabinoid 2 receptor in the development of bone cancer pain. Zhonghua Yi Xue Za Zhi.

[88] de Almeida AS (2019). Characterization of cancer-induced nociception in a murine model of breast carcinoma. Cell. Mol. Neurobiol..

[89] Guerrero AV, Quang P, Dekker N, Jordan RCK, Schmidt BL (2008). Peripheral cannabinoids attenuate carcinoma-induced nociception in mice. Neurosci. Lett..

[90] Saghafi N, Lam DK, Schmidt BL (2011). Cannabinoids attenuate cancer pain and proliferation in a mouse model. Neurosci. Lett..

[91] Lu C (2017). A Single intrathecal or intraperitoneal injection of cb2 receptor agonist attenuates bone cancer pain and induces a time-dependent modification of GRK2. Cell. Mol. Neurobiol..

[92] Ji D, Liang Z, Liu G, Zhao G, Fang J (2017). Bufalin attenuates cancer-induced pain and bone destruction in a model of bone cancer. Naunyn Schmiedebergs Arch. Pharmacol..

[93] Jiang W, Wang Y, Sun W, Zhang M (2017). Morin suppresses astrocyte activation and regulates cytokine release in bone cancer pain rat models. Phytother. Res..

[94] Anderson SP, Zylla DM, McGriff DM, Arneson TJ (2019). Impact of medical cannabis on patient-reported symptoms for patients with cancer enrolled in Minnesota's Medical Cannabis Program. J. Oncol. Pract..

[95] Aviram J (2020). Short-term medical cannabis treatment regimens produced beneficial effects among palliative cancer patients. Pharmaceuticals (Basel).

[96] Bar-Lev Schleider L (2018). Prospective analysis of safety and efficacy of medical cannabis in large unselected population of patients with cancer. Eur. J. Intern. Med..

[97] Bar-Sela G (2013). The medical necessity for medicinal cannabis: prospective, observational study evaluating the treatment in cancer patients on supportive or palliative care. Evid. Based Complement. Altern. Med..

[98] Bar-Sela G, Zalman D, Semenysty V, Ballan E (2019). The effects of dosage-controlled cannabis capsules on cancer-related cachexia and anorexia syndrome in advanced cancer patients: Pilot study. Integr. Cancer Ther..

[99] Calcaterra SL (2020). A population-based survey to assess the association between cannabis and quality of life among colorectal cancer survivors. BMC Cancer.

[100] Davies BH, Weatherstone RM, Graham JD, Griffiths RD (1974). A pilot study of orally administered Δ(1)-trans-tetrahydrocannabinol in the management of patients undergoing radiotherapy for carcinoma of the bronchus. Br. J. Clin. Pharmacol..

[101] Elliott DA, Nabavizadeh N, Romer JL, Chen Y, Holland JM (2016). Medical marijuana use in head and neck squamous cell carcinoma patients treated with radiotherapy. Support. Care Cancer.

[102] Good PD, Greer RM, Huggett GE, Hardy JR (2020). An open-label pilot study testing the feasibility of assessing total symptom burden in trials of cannabinoid medications in palliative care. J. Palliat. Med..

[103] Grimison P (2020). Oral THC:CBD cannabis extract for refractory chemotherapy-induced nausea and vomiting: A randomised, placebo-controlled, phase II crossover trial. Ann. Oncol..

[104] Chapman S (2021). Medical cannabis in pediatric oncology: A survey of patients and caregivers. Support Care Cancer.

[105] Côté M, Trudel M, Wang C, Fortin A (2016). Improving quality of life with nabilone during radiotherapy treatments for head and neck cancers: A randomized double-blind placebo-controlled trial. Ann. Otol. Rhinol. Laryngol..

[106] Giustina A, Mazziotti G, Canalis E (2008). Growth hormone, insulin-like growth factors, and the skeleton. Endocr. Rev..

[107] Strewler GJ (2000). The physiology of parathyroid hormone-related protein. N. Engl. J. Med..

[108] Dehghan F (2014). The effect of relaxin on the musculoskeletal system. Scand. J. Med. Sci. Sports.

[109] Siclari VA, Guise TA, Chirgwin JM (2006). Molecular interactions between breast cancer cells and the bone microenvironment drive skeletal metastases. Cancer Metastasis. Rev..

[110] Elefteriou F (2018). Impact of the autonomic nervous system on the skeleton. Physiol. Rev..

[111] Mlost J, Bryk M, Starowicz K (2020). Cannabidiol for pain treatment: Focus on pharmacology and mechanism of action. Int. J. Mol. Sci..

[112] Donahue HJ (2000). Gap junctions and biophysical regulation of bone cell differentiation. Bone.

[113] Martin TA (2014). The role of tight junctions in cancer metastasis. Semin. Cell. Dev. Biol..

[114] Itzstein C, Coxon FP, Rogers MJ (2011). The regulation of osteoclast function and bone resorption by small GTPases. Small GTPases.

[115] Sharir H (2012). The endocannabinoids anandamide and virodhamine modulate the activity of the candidate cannabinoid receptor GPR55. J. Neuroimmune Pharmacol..

[116] Rossi F (2014). The genetic ablation or pharmacological inhibition of TRPV1 signalling is beneficial for the restoration of quiescent osteoclast activity in ovariectomized mice. Br. J. Pharmacol..

[117] Rossi F (2015). CB(2) and TRPV(1) receptors oppositely modulate in vitro human osteoblast activity. Pharmacol. Res..

[118] Weber LV (2016). Expression and functionality of TRPV1 in breast cancer cells. Breast Cancer.

[119] Wu TT, Peters AA, Tan PT, Roberts-Thomson SJ, Monteith GR (2014). Consequences of activating the calcium-permeable ion channel TRPV1 in breast cancer cells with regulated TRPV1 expression. Cell Calcium.

[120] Chien CS (2013). Dual effect of capsaicin on cell death in human osteosarcoma G292 cells. Eur. J. Pharmacol..

[121] Naziroglu M (2017). Targeting breast cancer cells by MRS1477, a positive allosteric modulator of TRPV1 channels. PLoS ONE.

[122] Ghosh AK, Basu S (2010). Fas-associated factor 1 is a negative regulator in capsaicin induced cancer cell apoptosis. Cancer Lett..

[123] Lau JK (2014). Capsaicin induces apoptosis in human small cell lung cancer via the TRPV6 receptor and the calpain pathway. Apoptosis.

[124] Chow J, Norng M, Zhang J, Chai J (2007). TRPV6 mediates capsaicin-induced apoptosis in gastric cancer cells–Mechanisms behind a possible new "hot" cancer treatment. Biochim. Biophys. Acta.

[125] Castillo-Arellano J, Canseco-Alba A, Cutler SJ, Leon F (2023). The polypharmacological effects of cannabidiol. Molecules.

