# Supplementary material for: Classical cannabinoid receptors as target in cancer-induced bone pain: a systematic review, meta-analysis and bioinformatics validation
Source: Sci Rep. 2024 Mar 9;14:5782. doi: 10.1038/s41598-024-56220-0 (PMC10924854; doi:10.1038/s41598-024-56220-0)
Supplement: Supplementary file 1 — Supplementary Information. [file 41598_2024_56220_MOESM1_ESM.docx]

**Zeng et. al. Classical Cannabinoid Receptors as Potential Target in Cancer-induced Bone Pain: A Systematic Review and Meta-Analysis**

**SUPPLEMENTARY MATERIALS**

**Content**

1. **Supplementary Tables**

**Table S1.** Search strings for PubMed, Web of Science and Scopus

**Table S2.** Characteristics and outcomes of animal studies.

**Table S3.** Characteristics and outcomes of human prospective studies.

**Table S4.** Characteristics of included human retrospective studies.

**Table S5.** Characteristics of included human observational cross-sectional studies.

**Table S6.** Summary of meta-analysis of included studies showing non-significant association of cancer related bone pain with pharmacological modulator of CB_1_ and/or CB_2_ receptors in animal models.

**Table S7.** Summary of meta-analysis of included studies showing non-significant association of cancer-related bone pain with pharmacological modulator of CB_1_ and/or CB_2_ receptors in humans.

**Table S8**. Adapted Newcastle-Ottawa Scale for quasi-experimental, prospective cohort, retrospective cohort and observational studies

**Table S9**. Quality assessment of studies using a modified Newcastle-Ottawa scale (NOS) for quasi-experimental, prospective cohort, retrospective cohort and observational studies.

**Table S10.** PRISMA checklist for article.

**Table S11.** PRISMA checklist for abstract.

1. **Supplementary figures**

**Figure S1.** Risk of bias (RoB) assessment for animal studies using the SYRCLE RoB tool.

**Figure S2.** Risk of bias (RoB) assessment for human studies using the revised Cochrane risk-of-bias tool (RoB 2) for randomized trials (RoB 2 tool)

**Figure S3.** Network of predicted associations for CB_1_ (*cnr1*) and/or CB_2_ (*cnr2*) gene mouse, rat, and human and in STRING database.

1. **Supplementary references**
2. **Supplementary tables:**

**Table S1.** Search strings for PubMed, Web of Science and Scopus.

| **PubMed (Medline)** | **Web of Science** | **Scopus** |
| --- | --- | --- |
| 1. Cannabis or cannabinol or cannabidiol or cannabinoid or cannabis sativa or cannabigerol or CBD or cannabi*).tw. 2. (Marijuana or marihuana).tw. 3. (9tetrahydrocannabinol or delta-9-THC or tetrahydrocannabinol or delta-9-tetrahydrocannabinol or D9-tetrahydrocannabinol or THC or tetrahydrocannabinol* or tetra-hydrocannabinol* or 9?tetrahydrocannabinol* or DELTA?9?- tetrahydrocannabinol*).tw. 4. Endocannabinoid*.tw 5. (Dronabinol or nabilone or marinol or levonantradol or ajulemic acid).mp. 6. (Sativex or nabiximol).mp. 7. (Pot or ganja or hemp or hashish or hash*).mp. 8. or/1-7 9. Cancer*.tw. 10. Neoplasm.tw. 11. Sarcoma.tw. 12. Carcino*.tw. 13. Adenocarcinoma.tw. 14. (metastas?s or metastasis or metasta*).mp. 15. Tumo?r.tw. 16. Malignan*.tw. 17. Precancerous.tw. 18. Neoplasia.tw. 19. Onco*.tw. 20. Tumo?rigenesis.tw. 21. or/9-20 22. Pain.tw. 23. chronic pain.tw. 24. neuropathic pain.tw. 25. cancer pain.tw. 26. analgesi*.tw. 27. somatic pain.tw. 28. visceral pain.tw. 29. bone pain.tw. 30. idiopathic pain.tw. 31. malignant pain.tw. 32. oncologic pain.tw. 33. or/22-32 34. 8 and 21 and 33 35. Review.pt. 36. 34 not 35 | 1. TS=(Cannabis or cannabinol or cannabidiol or cannabinoid or cannabis sativa or cannabigerol or CBD or cannabi*) 2. TS=(Marijuana or marihuana) 3. TS=(9tetrahydrocannabinol or delta-9-THC or tetrahydrocannabinol or delta-9-tetrahydrocannabinol or D9-tetrahydrocannabinol or THC or tetrahydrocannabinol* or tetra-hydrocannabinol* or 9?tetrahydrocannabinol* or DELTA?9?- tetrahydrocannabinol*) 4. TS=Endocannabinoid* 5. TS=(Dronabinol or nabilone or marinol or levonantradol or ajulemic acid) 6. TS=(Sativex or nabiximol) 7. TS=(Pot or ganja or hemp or hashish or hash*) 8. #1 or #2 or #3 or #4 or #5 or #6 or #7 9. TS=Cancer* 10. TS=Neoplasm 11. TS=Sarcoma 12. TS=Carcino* 13. TS=Adenocarcinoma 14. TS=(metastas?s or metastasis or metasta*) 15. TS=Tumo?r 16. TS=Malignan* 17. TS=Precancerous 18. TS=Neoplasia 19. TS=Onco* 20. TS=Tumo?rigenesis 21. #9 or #10 or #11 or #12 or #13 or #14 or #15 or #16 or #17 or #18 or #19 or #20 22. TS=Pain 23. TS=chronic pain 24. TS=neuropathic pain 25. TS=cancer pain 26. TS=analgesi* 27. TS=somatic pain 28. TS=visceral pain 29. TS=bone pain 30. TS=idiopathic pain 31. TS=malignant pain 32. TS=oncologic pain 33. #22 or #23 or #24 or #25 or #26 or #27 or #28 or #29 or #30 or #31 or #32 34. #8 and #21 and #33 35. (#34) AND DT=(Review) 36. #34 not #35   *DocType=All document types; Language=All languages* | 1. TITLE-ABS-KEY(Cannabis or cannabinol or cannabidiol or cannabinoid or cannabis sativa or cannabigerol or CBD or cannabi*) 2. TITLE-ABS-KEY(Marijuana or marihuana) 3. TITLE-ABS-KEY(9tetrahydrocannabinol or delta-9-THC or tetrahydrocannabinol or delta-9-tetrahydrocannabinol or D9-tetrahydrocannabinol or THC or tetrahydrocannabinol* or tetra-hydrocannabinol* or 9?tetrahydrocannabinol* or DELTA?9?- tetrahydrocannabinol*) 4. TITLE-ABS-KEY(Endocannabinoid*) 5. TITLE-ABS-KEY(Dronabinol or nabilone or marinol or levonantradol or ajulemic acid) 6. TITLE-ABS-KEY(Sativex or nabiximol) 7. TITLE-ABS-KEY(Pot or ganja or hemp or hashish or hash*) 8. #1 or #2 or #3 or #4 or #5 or #6 or #7 9. TITLE-ABS-KEY(Cancer*) 10. TITLE-ABS-KEY(Neoplasm) 11. TITLE-ABS-KEY(Sarcoma) 12. TITLE-ABS-KEY(Carcino*) 13. TITLE-ABS-KEY(Adenocarcinoma) 14. TITLE-ABS-KEY(metastas?s or metastasis or metasta*) 15. TITLE-ABS-KEY(Tumo?r) 16. TITLE-ABS-KEY(Malignan*) 17. TITLE-ABS-KEY(Precancerous) 18. TITLE-ABS-KEY(Neoplasia) 19. TITLE-ABS-KEY(Onco*) 20. #9 or #10 or #11 or #12 or #13 or #14 or #15 or #16 or #17 or #18 or #19 or #20 21. TITLE-ABS-KEY(Pain) 22. TITLE-ABS-KEY(“chronic pain”) 23. TITLE-ABS-KEY(“neuropathic pain”) 24. TITLE-ABS-KEY(“neuropathic pain”) 25. TITLE-ABS-KEY(“cancer pain”) 26. TITLE-ABS-KEY(analgesi*) 27. TITLE-ABS-KEY(“somatic pain”) 28. TITLE-ABS-KEY(“visceral pain”) 29. TITLE-ABS-KEY(“bone pain”) 30. TITLE-ABS-KEY(“idiopathic pain”) 31. TITLE-ABS-KEY(“malignant pain”) 32. TITLE-ABS-KEY(“oncologic pain”) 33. #22 or #23 or #24 or #25 or #26 or #27 or #28 or #29 or #30 or #31 or #33 34. #8 and #21 and #34 35. #34 AND (LIMIT-TO (DOCTYPR, “ar”) OR (LIMIT-TO (DOCTYPR, “cp”) OR LIMIT-TO (DOCTYPR, “sh”)) |

Search strategies for the databases “Medline”, “Web of Science” and “Scopus”, respectively. The search strategies described include all the keywords used to derive to the total of 1778 papers that were used in this research to conduct a meta-analysis.

**Table S2.** Characteristics and outcomes of animal studies

| **Study** | **Species** | **Interventions** | **Model** | **Method of analyzing study outcomes** | **Funding source** |
| --- | --- | --- | --- | --- | --- |
| Ma, C., et al., 2021**^1^** | Rats | AM1241 | After intrathecal catheterization, each rat was implanted with Walker 256 tumour cells (provided by the Institute of Materia Medica, Chinese Academy of Medical Sciences, China) on the plantar region of the right hindpaw. | Paw mechanical withdrawal threshold (g) (PMWT) to von Frey filaments in the up-down method 5 times at 5-seconds intervals. Paw withdrawal thermal latency (s) (PWTL) tested by unilateral hot plate (UHP) test. | National Natural Science Foundation of China [grant numbers 8177051154, 81601150]; the Nn10 Program of Harbin Medical University Cancer Hospital; the China Postdoctoral Science Fund [grant number 2018M641844]; and the Haiyan Research; Fund of Cancer Hospital of Harbin Medical University [grant number JJQN2018-09]. |
| Wang, C., et al., 2020**^2^** | Mice | JWH015 | 20 μL of α–Minimum Essential Medium (α-MEM) (Thermo Fisher Scientific, USA) containing 2×10^5^ NCTC 2472 cells or α-MEM without cells were injected into the intramedullary cavity of the right distal femur, and designated the tumour group of mice and sham group of mice, respectively. | Paw mechanical withdrawal threshold (g) (PMWT) to von Frey filaments (0.16, 0.4, 0.6, 1.0, 1.4 and 2.0 g; Stoelting, USA). Numbers of spontaneous flinches (/2mins) was quantified over 2 min after a 30 min acclimation period. | The study was supported by the National Natural Science Foundation of China (Nos 81671087, 81971044, 81870871, 81701102, 81771142). |
| Mao, Y., et al., 2019**^3^** | Mice | JWH015 | 20 μl α‑minimum essential medium (Thermo Fisher Scientific, Inc.) containing 2×10^5^ NC TC 2472 osteolytic sarcoma cells was injected into the intramedullary space of the right femur. | Paw mechanical withdrawal threshold (g) (PMWT) to von Frey filaments (0.16, 0.4, 0.6, 1.0, 1.4 and 2.0 g; Stoelting, USA). Numbers of spontaneous flinches (/2mins) was quantified over 2 min after a 30 min acclimation period. | The present study was supported by The National Natural  Science Foundation of China (grant. nos. 81471129, 81671087,  81500954, 81701102 and 81771142) and a grants from  The Department of Health of Jiangsu Province of China  (grant. nos. XK101140 and RC 2011006). |
| Zhang, M., et al., 2017**^4^** | Rats | AM630  AM1241 | After a 1-week habituation period, animals received Walker 256 breast carcinoma cell implantation on the plantar region of the right hind paw of each rat. | Paw withdrawal thermal latency (s) (PWTL) tested by unilateral hot plate (UHP) test. | The present study was supported by the Translation Medicine Special Foundation of China Russia Medical Research Centre (grant nos. 201519 and CR1418), Nature Science Foundation of China (grant no. 81571885) and Haiyan Research Fund of Cancer Hospital of Harbin Medical University (grant no. JJQN2014-09). |
| Lu, C., et al., 2017**^+5^** | Rats | JWH015 | A 23-gauge needle was inserted to perforate the bone cortex and 5-μl volume of Walker 256 rat mammary gland carcinoma cells (1×10^5^/μl) or normal saline was injected into the intramedullary space using a 25-ll microsyringe. | Paw mechanical withdrawal threshold (g) (PMWT) to von Frey filaments (Stoelting, Wood Dale, IL, USA). The extent of ipsilateral limb use was observed for 2 min during spontaneous ambulation and ambulatory pain scores were characterized using the following criteria: score 0, normal use: score 1, slight limp; score 2, limp and guarding behavior; score 3, severe limp or partial non-use of the limb in locomotor activity; and score 4, complete lack of the limb use. | This research was supported by the National  Natural Science Foundation of China (81371207, 81070892,  81171048 and 81171047) and a grant from the Department of Health  of Jiangsu Province of China (XK201140, RC2011006). |
| Ji, D., et al., 2017**^6^** | Mice | Bufalin + AM630 | Bone cancer was established by inoculating Walker 256 cells (2×10^5^, 10 μL) into the intramedullary space of the mouse femur. | Paw withdrawal thermal latency (s) (PWTL) tested by hot plate test. | None. |
| Jiang, W., et al., 2017**^7^** | Rats | Morin + AM630 | Five-microliter Walker 256 rat mammary gland carcinoma cells (1 ×10^5^/μL) were injected into the left tibia cavity. | 50% paw withdrawal thresholds to von Frey filaments (Stoelting, Wood Dale, IL, USA). | None. |
| Gonzalez-Rodriguez, S., et al., 2017**^8^** | Mice | AM1241  JWH133  ACEA | A suspension of 10^5^ cells in 5 μL of PBS was injected into the right tibial medullar cavity and next, acrylic glue (Hystoacril®, Braun) was applied on the tibial plateau incised area. | Paw withdrawal thermal latency (s) (PWTL) tested by unilateral hot plate (UHP) test. Mechanical threshold values were obtained by performing the von Frey test. | Grants for pharamacology (University de Oviedo, Spain) were provided by Foundation for Scientific and Technical Research de Asturias and FEDER (European Union) (FC-15-GRUPIN13-125). S.G.-R is recipient of a postdoctoral grant from Clarin Program (Asrutias)-Marie Curie-Co fund. IUOPA is supported by Foundation Banking Caja de Ahorros de Asturias (Asturias, Spain). |
| Zhang, M., et al., 2016**^9^** | Rats | AM630  AM1241 | Each animal received Walker 256 tumour cell implantation on the plantar region of the right hindpaw. | Paw mechanical withdrawal threshold (g) (PMWT) to von Frey filaments. Paw withdrawal thermal latency (s) (PWTL) tested by unilateral hot plate (UHP) test. | This research was supported by funds from the Translational  Medicine Special Foundation of China Russia Medical Research Center (no. 201519 and CR1418) and the Technological and Innovative Talent Foundation of Harbin (2012RFXXS041). |
| Lu, C., et al., 2015**^10^** | Rats | JWH015 | A 23-gauge needle was used to perforate the bone cortex, and 5 μl Walker 256 rat mammary gland carcinoma cells (1×10^5^/ μl) were injected into the left tibia cavity using a 25-μl microsyringe. | Paw mechanical withdrawal threshold (g) (PMWT) to von Frey filaments (Stoelting, Wood Dale, IL, USA). The extent of ipsilateral limb use was observed for 2 min during spontaneous ambulation and ambulatory pain scores were characterized using the following criteria: score 0, normal use: score 1, slight limp; score 2, limp and guarding behavior; score 3, severe limp or partial non-use of the limb in locomotor activity; and score 4, complete lack of the limb use. | This research was supported by the National Natural  Science Foundation of China (81070892, 81171047,  81171048, and 81371207) and a grant from the Department  of Health of Jiangsu Province of China (XK201140,  RC2011006). |
| Lozano-Ondoua, A.N., et al., 2013**^11^** | Mice | JWH015  AM1241  SR144528  SR141716 | The condyles of the right distal femoris were exposed and a hole was drilled to create a space for injection of 1×10^5^ 66.1 cells in 5 μL complete MEM or 5 μL complete MEM without cells in control animals within the intramedullary space of the mouse femoris. | Paw mechanical withdrawal threshold (g) (PMWT) to von Frey filaments using the Chalplan up-down method. Numbers of flinches and guarding in 2 mins observed during a resting state. | This work was supported by NIH grants R01 CA142115-01, the AZCC Better Than Ever grant, and the Maine Cancer Foundation. |
| Cui, J.H., J. Ju, and M.H. Yoon, 2013**^12^** | Rats | ACEA  AM1241 | Syngeneic MRMT-1 rat mammary gland carcinoma cells were cultured in media and then media or 1×10^5^ tumour cells were injected into the medullary cavity of the right tibia to induce bone tumour. | Paw mechanical withdrawal threshold (g) (PMWT) to von Frey filaments (0.4-15g; Stoelting, USA). | NA |
| Uhelski, M.L., et al., 2013**^13^** | Mice | WIN 55,212-2 | Cells were then counted via hemocytometer pelletted, re-suspended and rinsed in PBS, pelletted a second time, then re-suspended in PBS for implantation at a concentration of 2×10^5^ 2472 cells/10 μl. Mice were briefly anesthetized using isoflurane (2–3%) and cancer cells were injected unilaterally into and around the calcaneous bone of the left hind paw ing a 29 g needle. | Mechanical response thresholds were obtained using a set of calibrated von Frey monofilaments. | This work was supported by NIH grants DA011471 and CA091007. Megan Uhelski was supported by NIDA training grant T32 DA07234. |
| Wang, D., et al., 2012***^14^** | Mice | JWH015 | 20 μL of α–Minimum Essential Medium (α-MEM) (Thermo Fisher Scientific, USA) containing 2×10^5^ NCTC 2472 cells or α-MEM without cells were injected into the intramedullary cavity of the right distal femur, and designated the tumour group of mice and sham group of mice. | Paw withdrawal mechanical threshold (g) (PWMT) and paw withdrawal thermal latency (s) (PWTL), details were not mentioned. | NA. |
| Khasabova, I.A., et al., 2011**^15^** | Mice | AM1241  ACPA | Under isoflurane (2%) anesthesia, NCTC clone 2472 fibrosarcoma cells (2×10^5^ cells in 10 μl of phosphate buffered saline, pH 7.3) were injected into and around the calcaneus bone of the animal’s left hind paw. | Paw withdrawal frequency (%) to von Frey monofilaments. | This work was supported by grants from the National Institute for Drug Abuse (DA011471, DAS) and the National Cancer Institute [CA091007 (DAS), CA138684 (VSS)]. The authors are grateful to P. Villalta and the University of Minnesota Cancer Center for assistance in the quantification of endocannabinoids. |
| Khasabova, I.A., et al., 2011**^16^** | Mice | 2-AG  AM630 | NCTC clone 2472 fibrosarcoma cells (2×10^5^ cells in 10 μl of phosphate buffered saline, pH 7.3) were injected into and around the calcaneus bone of the animal’s left hind paw while the mouse was anesthetized with isoflurane (2%). | Paw withdrawal frequency (%) to von Frey monofilaments. | Sources of support: This work was supported by grants from the National Institute for Drug Abuse (DA011471, DAS) and the National Cancer Institute [CA091007 (DAS), CA138684 (VSS)]. |
| Gu, X., et al., 2011**^17^** | Mice | JWH015 | Mice were briefly anesthetized with pentobarbital (50 mg/kg), and 20L-MEM containing no or 2×10^5^ NCTC2472 cells was injected into the femoral bone of the right hindpaw using a 25-L syringe. | Paw mechanical withdrawal threshold (g) (PMWT) to von Frey filaments. Paw withdrawal thermal latency (s) (PWTL) tested by hot plate test. | NA |
| Cui, J.H., et al., 2011**^18^** | Rats | WIN 55,212-2 | 20μl of culture medium or tumour cells were injected into the intramedullary cavity with a hand-driven, gear-operated syringe pump. | Paw mechanical withdrawal threshold (g) (PMWT) to von Frey filaments. | This study was supported by a grant (CRI-09039-1) Chonnam  National University Hospital Research Institute of Clinical  Medicine. |
| Saghafi, N., D.K. Lam, and B.L. Schmidt, 2011***^19^** | Mice | WIN 55,212-2  ACEA  AM1241 | The oral cancer mouse model was produced by inoculating HSC3 cancer cells into the hindpaw of mice. | Mechanical threshold change (%of basline) to an electronic von Frey anesthesiometer (IITC Life Sciences, Woodland Hills) after thirty minutes was allowed for acclimatization. | This work was supported by PACCTR summer fellowship, NIH/NCRR/OD UCSF-CTSI Grant Number TL1 RR024129 and NIH/NIDCR R21 DE018561. We thank S.H. Achdjian and D. Dang for technical assistance. |
| Lozano-Ondoua, A.N., et al., 2010**^20^** | Mice | AM1241 | The condyles of the right distal femur were exposed and a hole was drilled to create a space for a needle injection of 25,000 CCL-11 (NCTC clone 2472) murine sarcoma cells in 5 uL of alpha minimal essential medium containing 1% bovine serum albumin or 5 uL of alpha minimal essential medium alone (control) within the intramedullary space of the mouse femur. | Paw mechanical withdrawal threshold (g) (PMWT) to von Frey filaments. Numbers of flinches and guarding in 2 mins observed during a resting state. | NA |
| Curto-Reyes, V., et al., 2010**^21^** | Mice | AM1241  SR144528 | A minimal skin incision was made in the right leg exposing the tibial plateau and a 22 gauge needle coupled to a Hamilton syringe with 10^5^ NCTC 2472 or B16-F10 cells suspended in 5 μL of PBS was used to inject the cells into the medullar cavity. | 50% paw withdrawal threshold (g) to a von Frey test. Paw withdrawal thermal latency (s) (PWTL) tested by unilateral hot plate (UHP) test. | Grants were provided by MEC-FEDER (SAF2009-10567).  SR144528 was kindly donated by Sanofi-Aventis, France. VC-R  received a grant from Gobierno del Principado de Asturias  (Plan de Ciencia, Tecnología e Innovación de Asturias 2006–  2009). SL received a grant from CIBER of Enfermedades Raras  of the Instituto de Salud Carlos III (ISCIII). The Instituto  Universitario de Oncología is supported by Obra Social  Cajastur-Asturias, Spain. |
| Furuse, S., et al., 2009**^22^** | Mice | ACEA  AM630  AM251 | Either 20 μl of α-minimum essential medium (sham-implanted mice) or 20 μl of medium containing 1×10^5^ sarcoma cells (sarcoma-implanted mice) was injected using a 29-gauge needle and a 0.25-ml syringe. | Numbers of flinches and guarding in 2 mins observed during a resting state. Time on bar tested via bar test. | NA |
| Potenzieri, C., C. Harding-Rose, and D.A. Simone, 2008**^23^** | Mice | WIN 55,212-2 | Mice were placed in an enclosed chamber and anesthetized with 2% halothane, and fibrosarcoma cells (2×10^5^/10 μl PBS) were injected unilaterally into and around the calcaneus bone in each animal’s left hind paw using a 0.3ml insulin syringe. | Paw withdrawal frequency (%) to von Frey monofilaments. Time on bar tested via bar test. | This work was supported by grants from the National Institutes of Health (CA91007 and DA011471 to D.A.S). C.P was supported by training grant from the National Institute on Drug Abuse (5T32- DA007234). |
| Khasabova, I.A., et al., 2008**^24^** | Mice | AM281  AEA | Fibrosarcoma cells (2×10^5^) were injected unilaterally into and around the calcaneus bone of mice in 10μl of PBS, pH 7.3, under isoflurane anesthesia. | Paw withdrawal frequency (%) to von Frey monofilaments. | NA |
| Guerrero, A.V., et al., 2008**^25^** | Mice | WIN 55,212-2  AM1241 | SCC injections consisted of 1×10^6^ tumour cells in 50 μl of Dulbeco's modified Eagle's medium (DMEM) into the plantar surface of the right hind paw. | Mean percentage of pressure to elict paw withtrawal (std error bars), paw withdrawal thresholds (in text, no value of control). | This work was supported by Tobacco-related disease research program grants 12KT-0166 and NIH/NIDCR DE14609, T32-DE07306-11. |
| Hald, A., et al., 2007***^26^** | Mice | WIN 55,212-2 | A hole was drilled between the condyles to the marrow cavity with a 30 gauge needle and 100,000 NCTC-2472 cells in 10 µl α-MEM were injected. | Weight bearing (%), threshold (g), time on rotarod, Moltility counts, limb use day 14, 19 score. | NA |
| Hamamoto, D.T., S. Giridharagopalan, and D.A. Simone, 2007**^27^** | Mice | CP 55,940 | Mice were briefly anesthetized with halothane (2–3%) and fibrosarcoma cells (2×10^5^ cells/10 μl) were injected into and around the calcaneous bone of the left hind paw using a 0.3 cc insulin syringe with a 29.5 gauge needle. | Paw withdrawal frequency (%) to von Frey monofilaments (Stoelting Co, Wood Dale, IL). Time on bar tested via bar test. | This study was supported by grants from the National Institutes of Health; DA18231 (DTH), DA11471 and CA91007 (DAS). |
| Kehl, L.J., et al., 2003**^28^** | Mice | WIN 55,212-2 | Tumour cells (2×10^5^ in 10 μl PBS/humerus) were implanted into the medullary canals of both humeri | Time on bar tested via bar test. Tumour-evoked hyperalgesia measured by forelimb grip force (g). | This research was supported by grants from the National Institutes of Health (DA11471 and CA91007 to D.A.S). D.L.C. and B.D.N. were supported by training grant 2T35-DE07098. |
| de Almeida, A.S., et al., 2019^29^ | Mice | WIN 55,212-2 | 4T1 tumour cells were resuspended in phosphate buffer and 50 µl of the cell suspension or vehicle (phosphate buffer) were injected into the right fourth mammary fat pad of female BALB/c mice (10^4^ cells). | The mechanical sensitivity of animals was evaluated using von Frey filaments of increasing intensity (0.07–2 g). 50% paw withdrawal threshold (g) to a von Frey test (0.07, 0.16, 0.4, 0.6, 1.0, 1.4 and 2.0 g), with the results obtained, the value corresponding to 50% of the threshold, in grams, that each animal supports (threshold 50%) was calculated. | National Research Council of Brazil (CNPq; #422376/2016-  7) supported this work. |

**Table S3.** Characteristics and outcomes of human prospective studies (RCT, crossover or, quasi-experimental or cohort studies)

| **Study (author/year)** | **Study question/aim** | **Study design** | **Total number of patients** | **Intervention** | **Comparator** | **Duration**  **of treatment** | **Study results (relevant to pain)** | **Risk of bias** | **Funding source** |
| --- | --- | --- | --- | --- | --- | --- | --- | --- | --- |
| Davies, B.H., et al., 1974^30^ | To establish which aspects of therapy with  Δ^9^-THC predominate in naive patients in a  hospital environment, in a blind trial. | Crossover | 12 | Synthetic Δ^1^ THC: 10 mg | Non specified | 7 days | Patients were 'entirely free from pain when receiving THC. | Some concerns | No specified |
| Grimison. P., et al., 2020 ^31^**^+^** | To evaluate an  oral Δ^9^-THC:CBD for prevention of refractory chemotherapy-induced  nausea and vomiting. | Crossover | 68 | Δ^9^-THC 2.5 mg/CBD 2.5 mg | Non specified | 120 hours | The addition of oral THC:CBD to standard antiemetics was associated with less nausea and vomiting but additional side-effects. | Some concerns | The Department of Health, NSW Government, Australia. Tilray supplied and covered the cost of study treatments and were given the opportunity to review the study protocol and manuscript, but had no role in data analysis. |
| Jochimsen, P. R., 1978 ^32^ | To establish the effect of Benzopyranoperidine on pain | Crossover | 35 | Benzopyranoperidine, a Δ−^9^ -THC congener: 2, 4 mg | Codeine  sulfate: 60, 120 mg | 5 consecutive  days. | Benzopvranoperidine (2 or 4 mg) is not as effective as codeine (120 mg or 60 mg) and no more effective than placebo in relieving pain due to cancer. | Some concerns | Non specified |
| Noyes, R. Jr., et al., 1975 ^33^ | To establish the analgesic properties of Δ^9^-THC and codeine | Crossover | 34 | Δ^9^ -THC: 10 and 20 mg | Codeine:60, 120 mg | 7 hours | THC, 10 mg, was well tolerated and, despite its sedative effect, may have analgesic potential. | Some concerns | Non specified |
| Noyes, R. Jr., et al., 1975 ^34^ | To establish the analgesic Effect of Δ^9^-THC | Crossover | 10 | Δ^9^-Tetrahydrocannabinol: 5, 10, 15, and 20 mg | Non specified | 6 hours | Oral Δ^9^-THC (15 and 20 mg) reduced pain significantly compared to placebo. | Some concerns | Non specified |
| Staquet, M., et al., 1978 ^35^ | To establish the effect of a nitrogen analogue of Δ^9^-THC on cancer pain | Crossover | 26 | NIB: 4 mg | Trial 1: Codeine: 50 mg  Trial 2: Secobarbital: 50 mg | 6 hours | Trial 1: NIB was superior to placebo and approximately equivalent to 50 mg of codeine phosphate.  Trial 2: the tetrahydrocannabinol analog was superior to placebo and to 50 mg secobarbital. NIB is not useful clinically because of the frequency of side effects. | Some concerns | Non specified |
| Fallon, M.T., et al., 2017 ^36^ | To assess the analgesic efficacy of adjunctive Sativex (Δ^9^-THC (27 mg/mL): cannabidiol  (25 mg/mL)) in advanced cancer patients with chronic pain unalleviated by optimized opioid therapy. | RCT | Study 1: 399 patients.  Study 2: 540 patients. | Sativex oral  mucosal spray (THC (27 mg/mL): CBD (25 mg/mL)) | THC dosage non specified. | 35 Days | Sativex did not demonstrate superiority to placebo in reducing self-reported pain NRS scores in advanced cancer patients with chronic pain unalleviated by optimized opioid therapy. | Low risk | Otsuka Pharmaceutical Development & Commercialization, Inc., Rockville, MD, USA. |
| Johnson, J. R., et al., 2010 ^37^ | To compared the efficacy of Δ9-THC:CBD,  Δ^9^-THC with placebo,  in relieving pain in patients with advanced cancer. | RCT | 177 | THC (2.7 mg):CBD (2.5 mg) extract | THC extract: 2.7mg | 7 days | Mean pain Numerical Rating Scale (NRS) score was statistically significantly in favor of THC:CBD compared with placebo, whereas the THC group showed a nonsignificant  change. | Low risk | GW Pharma Ltd., Cambridgeshire, UK. |
| Turcott J.G., et al., 2018 ^38^ | To establish the effect of nabilone on appetite, nutritional status, and quality of life in lung cancer patients | RCT | 75 | Nabilone: 0.5 and 1 mg | Non specified | 2 weeks for 0.5 mg and 6 weeks for 1 mg | Nabilone is an adequate and safe therapeutic option to aid in the treatment of patients diagnosed with anorexia.  Larger trials are necessary in order to draw robust conclusions in regard to its efficacy in lung cancer patients. | Some concerns | Nabilone and placebo were donated by vealent pharmaceutical without any further participation in the trial. |
| Cote, M., 2015 ^39^ | To compare the effects of nabilone versus placebo on the quality of life and side effects during radiotherapy for head and neck carcinomas. | RCT | 56 | Nabilone  week1: 0.5 mg;  week 2: 1 mg;  week 3: 2 mg. | Non specified | 3 weeks | Nabilone was not potent enough to improve the patients’ quality of life over placebo. | Low risk | The Canadian Institutes of Health Research and the Fonds de recherche en santé du Québec. ICN Valeant Pharmaceuticals provided the nabilone and the placebo pills during the trial. |
| Lichtman A. H., et al., 2018 ^40^ | To assess adjunctive nabiximols (Sativex,  (Δ^9^-THC [27 mg/mL] and cannabidiol [25 mg/mL]), in advanced cancer patients with  chronic pain unalleviated by optimized opioid therapy. | RCT | 397 | Sativex : D9-tetrahydrocannabinol [27 mg/mL] and cannabidiol [25 mg/mL] | Non specified | 35 days | Nabiximols might have utility in patients with advanced cancer who receive a lower opioid dose. | Low risk | Otsuka Pharmaceutical Development & Commercialization, Inc., Rockville, MD, USA. The efforts of A.H. Lichtman were supported by the Virginia Commonwealth University School of Pharmacy start-up funds. |
| Portenoy, R. K., et al., 2012 ^41^ | To evaluate the analgesic efficacy and safety of Nabiximols in 3 dose ranges. | RCT | 360 | Nabiximols: 2.7 mg THC, 2.5 mg CBD | Non specified | 35 days | Nabiximols may be a useful add-on analgesic for patients with opioid-refractory cancer pain. | Low risk | GW Pharma Ltd., Cambridgeshire, UK, and Otsuka. |
| Zylla, D. M., et al., 2021 ^42^ | To assess the addition of medical cannabis to standard oncology care. | RCT | 30 | THC/CBD (2-30 mg and 5 - 40 mg). | Non specified | 15 days | The addition of MC to standard oncology care was well-tolerated and may lead to improved pain control and lower opioid requirements. | High risk | Health- Partners Institute; Randy Shaver Cancer Research and Community Fund; and philanthropic support from Diana and Bob Carter. Vireo Health and LeafLine Labs provided all cannabis products at no cost to the patients during the trial period. |
| Good P.D., et al., 2020 ^43^ | To assess the feasibility of using global symptom burden measures to assess response to medicinal cannabis. | RCT | 21 | CBD (CBD 100 mg/mL): 50 to 600 mg/day  THC (Delta-9-THC 10 mg/mL): 2.5 to 30 mg/day | Non specified | 28 days | THC and CBD were generally well tolerated and the outcome measure of total symptom distress is promising as a measure of overall symptom benefit. | High risk | NHMRC MRFF grant  APP1152232. |
| Tavhare, S. D., et al., 2019 ^44^ | To assess the analgesic potential of Jala- Prakshalana (Cannabis sativa L. leaves powder) in cancer patients. | Quasi-experimental | 24 | Cannabis leaf capsule: 200 mg, 3 time/day | Non specified | 4 weeks | Jalaprakshalana Shodhita Bhanga powder (250 mg thrice per day) relieves cancer-induced pain significantly and does not cause any major adverse effect and withdrawal symptoms during trial period. | Moderate risk (4/6 stars) | IPGT and RA, Gujarat Ayurved  University, Jamnagar for conducting this research as a part of  PhD research project of Dravyaguna department. |
| Bar-Sela G., et al., 2019^45^ | To evaluate the effect of dosage-controlled cannabis capsules on CACS in advanced cancer patients | Quasi-experimental | 24 | THC: 19 mg and CBD 1 mg | Non specified | 6 months | Improvement in appetite and mood as well as a reduction in pain and fatigue  was demonstrated. | Moderate risk (4/6 stars) | Cannabics Pharmaceuticals Inc, Bethesda, MD, USA. |
| Bar-Lev Schleider, L., et al., 2018 ^46^ | To characterize the epidemiology of cancer patients receiving medical cannabis treatment and describe safety and efficacy of this therapy. | Cohort | 2970 | Medical cannabis, dosage non specified. | Non specified | Non specified | Cannabis as a palliative treatment for cancer patients seems to be well tolerated, effective and safe option to help patients cope with the malignancy related symptoms. | Low risk (5/6 stars) | Tikun Olam Ltd. supported this study. |
| Bar-Sela G., et al., 2013 ^47^ | To evaluate the effect of cannabis as treatment of cancer patients on supportive or palliative Care | Cohort | 211 | Medical cannabis, dosage non specified. | Non specified | Non specified | The positive effects of cannabis on various cancer-related symptoms are tempered by reliance on self-reporting for many of the variables. The improvement in symptoms should push the use of cannabis in palliative treatment of oncology patients. | Moderate risk (4/6 stars) | Non specified |
| Aviram, J., et al., 2020 ^48^ | To assess the short-term outcomes of medicinal cannabis treatment prescribed by oncologists. | Cohort | 228 | Medical cannabis | Non specified | 6 months | THC-dominant treatments showed significant superiority in their beneficial effect only in sleep duration compared to CBD-dominant treatments. | Moderate risk (4/6 stars) | The Evelyn Gruss Lipper Charitable Foundation. |
| Kasvis, P., et al., 2022 ^49^ | TO explore whether symptom relief differs by sex in patients with cancer receiving medical cannabis. | Cohort | 358 | THC:CBD (2.5 mg THC: 1 mg CBD) | 2.5 mg THC alone | 3 months | CBD-dominant products did not offer significant symptom relief in either sex. | Low risk (5/6 stars) | The Canadian Consortium for the Investigation of Cannabinoids (CCIC), the Collège des Médecins du Québec (CMQ), and unrestricted grants from several licensed cannabis producers (Bedrocan, Mettrum, and Tweed; these three companies merged during the study conduct into one company called Canopy Growth Corporation). Cedars Cancer Foundation and Rossy Cancer Network. |
| Meghani, S. H., et al., 2021 ^50^ | To investigate how cannabis use influences pain relief. | Cohort | 136 | Cannabis use | Non specified | 5 months | In the absence of cannabis use, African Americans reported higher “least pain” scores compared to Whites | Low risk (8/9 stars) | An NIH/NINR award (R01NR017853), and National Research Service Award program (T32NR009356). |

Risk of bias for RCTs and crossover studies was assessed by the Cochrane risk-of-bias tool for randomized trials (RoB 2), whereas for quasi-experimental and cohort studies was assessed using the Newcastle – Ottawa Quality Assessment Scale. + RCT and crossover trial.

**Table S4.** Characteristics of included human retrospective studies.

| **Study (author/year)** | **Study question/aim** | **Study design** | **Number of patients assessed** | **Intervention** | **Length**  **of**  **follow-up (days)** | **Study results (relevant to pain)** | **Risk of bias** | **Funding source** |
| --- | --- | --- | --- | --- | --- | --- | --- | --- |
| Johnson, J. R., et al., 2013 ^51^ | To investigate the long-term safety and tolerability of THC/CBD spray and THC spray in relieving pain in patients with advanced cancer. | Retrospective cohort | 43 | THC:CBD (2.7 mg:2.5 mg) | Non specified | The efficacy end point of change from baseline in mean Brief Pain Inventory-Short Form scores for ‘‘pain severity’’ and ‘‘worst pain’’ domains showed a decrease (i.e., improvement) at each visit in the THC/CBD spray patients. | Moderate risk (4/6 stars) | GW Pharma Ltd., Cambridgeshire, UK. |
| Raghunathan, N. J., et al., 2022 ^52^ | To describe patient experiences with medical cannabis with focus on use contexts and patients’ reported benefits and harms. | Retrospective cohort | 163 | CBD and THC  use | Non specified | Among past CBD users, the most commonly reported benefits were less pain (21%). Among those with past THC use, reported benefits included improvement in pain (17%). | High risk (5/9 stars) | The National Institutes of Health/National Cancer Institute grant to Memorial Sloan Kettering Cancer Center (P30 CA008748), the Translational and Integrative Medicine Research Fund at Memorial Sloan Kettering Cancer Center, and the Herbal Education and Research in Oncology Program made possible by the Laurance S. Rockefeller Fund. |
| Wiseman L. K., et al., 2022 ^53^ | To determine if cannabis users have different pain  scores after gynaecologic oncology surgery than non-cannabis users. | Retrospective cohort | Cannabis use | 74 | 2 years | Pain scores were significantly higher after surgery in cannabis users than non-users. | High risk (5/9 stars) | Non specified |
| Pawasarat, I. M., et al., 2020 ^54^ | Retrospective chart review of medical cannabis-certified oncology patients was performed. | Retrospective cohort | 232 | Medical cannabis:  45 – 126 mg/day | Non specified | Pain, physical and total ESAS significantly  improved for medical cannabis (-) and medical cannabis (+); however, only medical cannabis (+) significantly improved emotional ESAS. | High risk (5/9 stars) | Non specified |
| Donovan, K.A., et al., 2020 ^55^ | To determine the rate of cannabis use in cancer patients, identify demographic and clinical correlates of use, and examine differences in moderate-to-severe symptoms between users and nonusers. | Retrospective cohort | 1243 | Cannabis use | Non specified | Cannabis use was associated with moderate-to-severe symptom- atology, including pain, | Moderate risk (7/9 stars) | Non specified |
| Donovan K.A., et al., 2019 ^56^ | To determine the extent to which patients seeking specialized symptom management were using cannabis and to compare the severity of cancer-related symptoms between users and nonusers. | Retrospective cohort | 816 | Cannabis use | Non specified | Cannabis use was not significantly associated with higher rates of moderate- to-severe pain. | Moderate risk (7/9 stars) | Non specified |
| Nathan, R., et al., 2022 ^57^ | To assess efficacy of medical cannabis for the treatment of pain, nausea, anorexia, insomnia and anxiety in elderly cancer patients. | Retrospective cohort | 83 | Medical cannabis use | 8-12 weeks | There was no statistically significant difference in pain between groups. | Low risk (5/6 stars) | The Roswell Park Cancer Institute and National Cancer Institute (NCI) grant P30CA016056. |
| Anderson, S. P., et al., 2019 ^58^ | To asses the impact of participating in the Minnesota medical cannabis program on managing symptoms in patients with cancer. | Retrospective cohort | 1120 | THC:CBD | 4 months | There was a significant reduction of medical cannabis in the severity of pain. | Low risk (5/6 stars) | Non specified |
| Waissengrin, B., et al., 2015 ^59^ | To analyse the indications for the administration of cannabis among adult Israeli cancer patients and evaluate its efficacy. | Retrospective cohort | 279 | Cannabis use | 6 months | Cannabis use improved pain. | Moderate risk (4/6 stars) | Non specified |
| Ofir, R., et al., 2019 ^60^ | To evaluate the effects of medical cannabis on alleviating side effects of anticancer treatment as an integral part of supportive and palliative care of children with cancer. | Retrospective cohort | 50 | Medical cannabis | 6 months | The effect of medical cannabis on pain was difficult to ascertain, since all the children continued to receive other analgesics along with their medical cannabis treatment. | Low risk (5/6 stars) | Non specified |

Risk of bias was using the Newcastle – Ottawa Quality Assessment Scale

**Table S5.** Characteristics of included human observational cross-sectional studies.

| **Study (author/year)** | **Study question/aim** | **Study design** | **Number of participants** | **Intervention** | **Delivery method of the survey** | **Self/non self-report** | **Study results (relevant to pain)** | **Risk of bias** | **Funding source** |
| --- | --- | --- | --- | --- | --- | --- | --- | --- | --- |
| Calcaterra, S. L., 2020 ^61^ | To determine the prevalence of cannabis use among colorectal cancer survivors and its associations with quality of life and cancer-related symptomatology. | Observational cross-sectional | 1784 | Cannabis use | Email survey | Self-reported | Cannabis users reported higher pain scores. | Moderate risk (7/9 stars) | The PORTAL (Patient Outcomes Research to Advance Learning) Network, a patient-Centered Outcomes Research Institute (PCORI) Award (CDRN-1306-04681 Phase II). |
| Chapman, S., et al., 2021 ^62^ | To characterize the prevalence and factors associated with the use of cannabis for the treatment of cancer and management of cancer-related symptoms in children during or after cancer treatment. | Observational cross-sectional | 64 | Medical cannabis | Questionary survey | Self-report | Cannabis users reported improved symptoms, particularly pain (10/14; 71.4%). | High risk (5/9 stars) | Non specified |
| Webster, E. M., et al., 2020 ^63^ | To evaluate patient experience with prescribed medical cannabis in women with gynecologic malignancies. | Observational cross-sectional | 31 | Medical cannabis | Single-institution survey | Self-report | Medical cannabis provided relief from cancer or treatment-related symptoms including bone pain. | Low risk (5/6 stars) | Non specified |
| Elliott, D. A., et al., 2016 ^64^ | To understand why patients with history of head and neck cancer treated with radiotherapy are using medical cannabis. | Observational cross-sectional | 15 | Medical cannabis | Questionary survey | Self-report | Medical cannabis provided benefit in pain relief. | Low risk (5/6 stars) | Non specified |

Risk of bias was using the Newcastle – Ottawa Quality Assessment Scale

**Table S6.** Summary of meta-analysis of included studies showing non-significant association of cancer-induced bone pain with pharmacological modulator of CB_1_ and/or CB_2_ receptors in animal models.

| **Intervention** | **Outcome** | **Gender (Species)** | **CB receptor (Strain, no. studies)** | **Groups** | **Subgroup (std.) mean difference (95%CI)** | **Overall (std.) mean difference (95% CI)** | **Statistical method** | **Test for heterogeneity** | **Test for overall effect** |
| --- | --- | --- | --- | --- | --- | --- | --- | --- | --- |
| **Synthetic agonist** | Paw withdrawal threshold (g) | Male  (Rats) | CB_2_-selective agonist (Wistar rats, 2) | 20 AM1241 (0.07 μg/day, 8 days), 20 Vehicle | NA | 6.29 [-2.85, 15.43] | Mean Difference (IV, Random, 95% CI) | Chi² = 34.23,  df = 1 (P < 0.00001);  I² = 97% | Z = 1.35  (p =0.18) |
| **Synthetic inverse**  **agonist** | Paw withdrawal frequency (%) | Male  (Mice) | CB_2_ inverse agonist (C3H/HeCr MTV-, 1)  CB_2_ inverse agonist (C3H/HeN, 1) | 6 AM630 (4 ug), 6 Vehicle  7 AM281 (1 ug), Vehicle | -1.91 [-13.66, 9.84]  11.55 [0.71, 22.39] | 5.02 [-8.17, 18.20] | Mean Difference (IV, Random, 95% CI) | Chi² = 2.72,  df = 1 (P = 0.10),  I² = 63.3% | Z = 0.75  (P = 0.46) |
|  | Paw withdrawal thermal latency (s) | Male  (Rats) | CB_2_ inverse agonist (Wistar rats, 2) | 20 AM630 (0.07 ug), 20 Vehicle | NA | 0.12 [-0.80, 1.04] | Mean Difference (IV, Fixed, 95% CI) | Chi² = 0.05, df = 1 (P = 0.82); I² = 0% | Z = 0.26  (P = 0.79) |

The analysis shows the mean difference or standard mean difference of different studies comparing the effects of the endocannabinoids AEA and 2-AG and verified CB_1/2_ selective synthetic agonists and inverse agonists on paw withdrawal frequency and thermal latency. Std., standardised; IV, inverse-variance weighting; NA, not applicable.

**Table S7.** Summary of meta-analysis of included studies showing non-significant association of cancer-related bone pain with pharmacological modulator of CB_1_ and/or CB_2_ receptors in humans.

| **Intervention** | **CB receptor** | **Number of studies** | **Outcome** | **N patients** | **Groups** | **Mean difference (95% CI)** | **Statistical method** | **Test for heterogeneity** | **Test for overall effect** |
| --- | --- | --- | --- | --- | --- | --- | --- | --- | --- |
| **THC:CBD extract, Sativex (Nabiximols)** | THC: partial agonist for CB_1_ and CB_2_ receptors  CBD: inverse agonist for CB_2_ receptor | 5 | NRS pain scores | 1304 | -60 THC:CBD extract, 59 placebo  -91 Nabiximols, 92 placebo  -200 Sativex, 199 placebo  -103 Sativex, 103 placebo  -199 Nabiximols, 198 placebo | -0.21 [-0.48, 0.07] | Mean Difference (IV, Random, 95% CI) | Chi² = 9.97, df = 4 (P = 0.04), I² = 60% | Z = 1.48 (P = 0.14) |
| **THC** | THC: partial agonist for CB_1_ and CB_2_ receptors | 2 | Pain reduction | 46 | 46 THC 10mg, 46 Placebo | 0.66 [-0.18, 1.49] | Mean Difference (IV, Fixed, 95% CI) | Chi² = 0.30, df = 1 (P = 0.59); I² = 0% | Z = 1.54 (P = 0.12) |

**Table S8.** Adapted Newcastle-Ottawa Scale for quasi-experimental, prospective cohort, retrospective cohort and observational studies

**NEWCASTLE - OTTAWA QUALITY ASSESSMENT SCALE**

Note: A study can be awarded a maximum of one star for each numbered item within the Selection and Outcome categories. A maximum of two stars can be given for Comparability

**Selection**

1) Representativeness of the exposed cohort

a) truly representative of the average in the community **🟑**

b) somewhat representative of the average in the community **🟑**

c) selected group of users eg nurses, volunteers

d) no description of the derivation of the cohort

2) Selection of the non exposed cohort

a) drawn from the same community as the exposed cohort **🟑**

b) drawn from a different source

c) no description of the derivation of the non exposed cohort

3) Ascertainment of exposure

a) secure record (eg surgical records) **🟑**

b) structured interview **🟑**

c) written self report

d) no description

4) Demonstration that outcome of interest was not present at start of study

a) yes **🟑**

b) no

**Comparability**

1) Comparability of cohorts on the basis of the design or analysis

a) study controls for *other drugs for pain management (e.g. opioids, morphine etc)***🟑**

b) study controls for *age and gender***🟑**

**Outcome**

1) Assessment of outcome

a) independent blind assessment **🟑**

b) record linkage **🟑**

c) self report

d) no description

2) Was follow-up long enough for outcomes to occur

a) yes (select an adequate follow up period for outcome of interest) **🟑**

b) no

3) Adequacy of follow up of cohorts

a) complete follow up - all subjects accounted for **🟑**

b) subjects lost to follow up unlikely to introduce bias (small number lost, or description provided of those lost) **🟑**

c) follow up rate adequate and no description of those lost

d) no statement

**NOTE: For single-arm studies with no comparator, the items highlighted in grey (‘*Selection of the non exposed cohort*’ and ‘*Comparability of cohorts on the basis of the design or analysis*’) were removed and not assessed. These studies were scored out of 6 stars instead of out of 9.**

**Table S9.** Quality assessment of studies using a modified Newcastle-Ottawa scale (NOS) for quasi-experimental, prospective cohort, retrospective cohort and observational studies.

| **Study ID** | **Selection** | | | | **Comparability** | **Outcomes** | | | **Total (out of 9** ☆**)** |
| --- | --- | --- | --- | --- | --- | --- | --- | --- | --- |
|  | **Representativeness of the exposed cohort (**☆**)** | **Selection of the non exposed cohort (**☆**)** | **Ascertainment of exposure (**☆**)** | **Demonstration that outcome of interest was not present at start of study (**☆**)** | **Comparability of cohorts on the basis of the design or analysis (**☆☆**)** | **Assessment of outcome (**☆**)** | **Was follow-up long enough for outcomes to occur (**☆**)** | **Adequacy of follow up of cohorts (**☆**)** |  |
| Anderson et al. 2019 | ☆ | NA | ☆ | ☆ | NA | / | ☆ | ☆ | ☆☆☆☆☆ (5)* |
| Aviram et al. 2020 | ☆ | NA | ☆ | ☆ | NA | / | ☆ | / | ☆☆☆☆ (4)* |
| Bar-Sela et al. 2013 | ☆ | NA | ☆ | ☆ | NA | / | ☆ | / | ☆☆☆☆ (4)* |
| Bar-Sela et al. 2019 | ☆ | NA | ☆ | ☆ | NA | / | ☆ | / | ☆☆☆☆ (4)* |
| Calcaterra et al. 2020 | ☆ | ☆ | ☆ | ☆ | ☆☆ | / | / | ☆ | ☆☆☆☆☆☆☆ (7) |
| Chapmann et al. 2021 | ☆ | ☆ | ☆ | ☆ | / | / | / | ☆ | ☆☆☆☆☆ (5) |
| Donovan et al. 2019 | ☆ | ☆ | ☆ | ☆ | ☆ | / | ☆ | ☆ | ☆☆☆☆☆☆☆ (7) |
| Donovan et al. 2020 | ☆ | ☆ | ☆ | ☆ | ☆ | / | ☆ | ☆ | ☆☆☆☆☆☆☆ (7) |
| Elliott et al. 2016 | ☆ | NA | ☆ | ☆ | NA | / | ☆ | ☆ | ☆☆☆☆☆ (5)* |
| Johnson et al. 2013 | ☆ | NA | ☆ | ☆ | NA | / | ☆ | / | ☆☆☆☆ (4)* |
| Kasvis et al. 2022 | ☆ | NA | ☆ | ☆ | NA | / | ☆ | ☆ | ☆☆☆☆☆ (5)* |
| Meghani et al. 2021 | ☆ | ☆ | ☆ | ☆ | ☆☆ | / | ☆ | ☆ | ☆☆☆☆☆☆☆☆ (8) |
| Nathan et al. 2022 | ☆ | NA | ☆ | ☆ | NA | / | ☆ | ☆ | ☆☆☆☆☆ (5)* |
| Ofir et al. (2019) | ☆ | NA | ☆ | ☆ | NA | / | ☆ | ☆ | ☆☆☆☆☆ (5)* |
| Pawasarat et al. 2020 | ☆ | ☆ | ☆ | ☆ | / | / | / | ☆ | ☆☆☆☆☆ (5) |
| Raghunathan et al. 2022 | ☆ | ☆ | ☆ | ☆ | / | / | / | ☆ | ☆☆☆☆☆ (5) |
| Schleider et al. 2018 | ☆ | NA | ☆ | ☆ | NA | / | ☆ | ☆ | ☆☆☆☆☆ (5)* |
| Tavhare et al. (2019) | ☆ | NA | ☆ | ☆ | NA | / | ☆ | / | ☆☆☆☆ (4)* |
| Waissengrin et al. (2015) | ☆ | NA | ☆ | ☆ | NA | / | ☆ | / | ☆☆☆☆ (4)* |
| Webster et al. 2020 | ☆ | NA | ☆ | ☆ | NA | / | ☆ | ☆ | ☆☆☆☆☆ (5)* |
| Wiseman et al. 2022 | ☆ | ☆ | ☆ | ☆ | / | / | / | ☆ | ☆☆☆☆☆ (5) |

**Table S10.** PRISMA checklist for article.

| **Section and Topic** | **Item #** | **Checklist item** | **Location where item is reported (page #)** |
| --- | --- | --- | --- |
| **TITLE** | | |  |
| Title | 1 | Identify the report as a systematic review. | 1 |
| **ABSTRACT** | | |  |
| Abstract | 2 | See the PRISMA 2020 for Abstracts checklist. | 2 |
| **INTRODUCTION** | | |  |
| Rationale | 3 | Describe the rationale for the review in the context of existing knowledge. | 3-4 |
| Objectives | 4 | Provide an explicit statement of the objective(s) or question(s) the review addresses. | 4 |
| **METHODS** | | |  |
| Eligibility criteria | 5 | Specify the inclusion and exclusion criteria for the review and how studies were grouped for the syntheses. | 5 |
| Information sources | 6 | Specify all databases, registers, websites, organisations, reference lists and other sources searched or consulted to identify studies. Specify the date when each source was last searched or consulted. | 4-5 |
| Search strategy | 7 | Present the full search strategies for all databases, registers and websites, including any filters and limits used. | Supplementary Table S1 |
| Selection process | 8 | Specify the methods used to decide whether a study met the inclusion criteria of the review, including how many reviewers screened each record and each report retrieved, whether they worked independently, and if applicable, details of automation tools used in the process. | 5-6 |
| Data collection process | 9 | Specify the methods used to collect data from reports, including how many reviewers collected data from each report, whether they worked independently, any processes for obtaining or confirming data from study investigators, and if applicable, details of automation tools used in the process. | 5-6 |
| Data items | 10a | List and define all outcomes for which data were sought. Specify whether all results that were compatible with each outcome domain in each study were sought (e.g. for all measures, time points, analyses), and if not, the methods used to decide which results to collect. | 5-6, Supplementary Tables S3-S5 |
|  | 10b | List and define all other variables for which data were sought (e.g. participant and intervention characteristics, funding sources). Describe any assumptions made about any missing or unclear information. | Supplementary Tables S3-S5 |
| Study risk of bias assessment | 11 | Specify the methods used to assess risk of bias in the included studies, including details of the tool(s) used, how many reviewers assessed each study and whether they worked independently, and if applicable, details of automation tools used in the process. | 6-7 |
| Effect measures | 12 | Specify for each outcome the effect measure(s) (e.g. risk ratio, mean difference) used in the synthesis or presentation of results. | Tables 2-4, Supplementary Tables S6-S7 |
| Synthesis methods | 13a | Describe the processes used to decide which studies were eligible for each synthesis (e.g. tabulating the study intervention characteristics and comparing against the planned groups for each synthesis (item #5)). | 5 |
|  | 13b | Describe any methods required to prepare the data for presentation or synthesis, such as handling of missing summary statistics, or data conversions. | 6 |
|  | 13c | Describe any methods used to tabulate or visually display results of individual studies and syntheses. | 6 |
|  | 13d | Describe any methods used to synthesize results and provide a rationale for the choice(s). If meta-analysis was performed, describe the model(s), method(s) to identify the presence and extent of statistical heterogeneity, and software package(s) used. | 6 |
|  | 13e | Describe any methods used to explore possible causes of heterogeneity among study results (e.g. subgroup analysis, meta-regression). | 6 |
|  | 13f | Describe any sensitivity analyses conducted to assess robustness of the synthesized results. | NA |
| Reporting bias assessment | 14 | Describe any methods used to assess risk of bias due to missing results in a synthesis (arising from reporting biases). | 6-7 |
| Certainty assessment | 15 | Describe any methods used to assess certainty (or confidence) in the body of evidence for an outcome. | 7 |
| **RESULTS** | | |  |
| Study selection | 16a | Describe the results of the search and selection process, from the number of records identified in the search to the number of studies included in the review, ideally using a flow diagram. | 7-8, Figure 1 |
|  | 16b | Cite studies that might appear to meet the inclusion criteria, but which were excluded, and explain why they were excluded. | NA |
| Study characteristics | 17 | Cite each included study and present its characteristics. | Supplementary Tables S3-S5 |
| Risk of bias in studies | 18 | Present assessments of risk of bias for each included study. | 8, Supplementary Tables S9, Supplementary Figures S1-S2 |
| Results of individual studies | 19 | For all outcomes, present, for each study: (a) summary statistics for each group (where appropriate) and (b) an effect estimate and its precision (e.g. confidence/credible interval), ideally using structured tables or plots. | Figure 2,  Table 2, Supplementary tables S6-S7 |
| Results of syntheses | 20a | For each synthesis, briefly summarise the characteristics and risk of bias among contributing studies. | 9-13 |
|  | 20b | Present results of all statistical syntheses conducted. If meta-analysis was done, present for each the summary estimate and its precision (e.g. confidence/credible interval) and measures of statistical heterogeneity. If comparing groups, describe the direction of the effect. | 9-13 |
|  | 20c | Present results of all investigations of possible causes of heterogeneity among study results. | NA |
|  | 20d | Present results of all sensitivity analyses conducted to assess the robustness of the synthesized results. | NA |
| Reporting biases | 21 | Present assessments of risk of bias due to missing results (arising from reporting biases) for each synthesis assessed. | NA |
| Certainty of evidence | 22 | Present assessments of certainty (or confidence) in the body of evidence for each outcome assessed. | 8-9 |
| **DISCUSSION** | | |  |
| Discussion | 23a | Provide a general interpretation of the results in the context of other evidence. | 15-18 |
|  | 23b | Discuss any limitations of the evidence included in the review. | 17-18 |
|  | 23c | Discuss any limitations of the review processes used. | 17-18 |
|  | 23d | Discuss implications of the results for practice, policy, and future research. | 15-17 |
| **OTHER INFORMATION** | | |  |
| Registration and protocol | 24a | Provide registration information for the review, including register name and registration number, or state that the review was not registered. | 20 |
|  | 24b | Indicate where the review protocol can be accessed, or state that a protocol was not prepared. | 20 |
|  | 24c | Describe and explain any amendments to information provided at registration or in the protocol. | NA |
| Support | 25 | Describe sources of financial or non-financial support for the review, and the role of the funders or sponsors in the review. | 19 |
| Competing interests | 26 | Declare any competing interests of review authors. | 20 |
| Availability of data, code and other materials | 27 | Report which of the following are publicly available and where they can be found: template data collection forms; data extracted from included studies; data used for all analyses; analytic code; any other materials used in the review. | 20 |

**Table S11.** PRISMA checklist for abstract.

| **Section and Topic** | **Item #** | **Checklist item** | **Reported (Yes/No)** |
| --- | --- | --- | --- |
| **TITLE** | | |  |
| Title | 1 | Identify the report as a systematic review. | Yes |
| **BACKGROUND** | | |  |
| Objectives | 2 | Provide an explicit statement of the main objective(s) or question(s) the review addresses. | Yes |
| **METHODS** | | |  |
| Eligibility criteria | 3 | Specify the inclusion and exclusion criteria for the review. | NO |
| Information sources | 4 | Specify the information sources (e.g. databases, registers) used to identify studies and the date when each was last searched. | YES |
| Risk of bias | 5 | Specify the methods used to assess risk of bias in the included studies. | NO |
| Synthesis of results | 6 | Specify the methods used to present and synthesise results. | YES |
| **RESULTS** | | |  |
| Included studies | 7 | Give the total number of included studies and participants and summarise relevant characteristics of studies. | YES |
| Synthesis of results | 8 | Present results for main outcomes, preferably indicating the number of included studies and participants for each. If meta-analysis was done, report the summary estimate and confidence/credible interval. If comparing groups, indicate the direction of the effect (i.e. which group is favoured). | YES |
| **DISCUSSION** | | |  |
| Limitations of evidence | 9 | Provide a brief summary of the limitations of the evidence included in the review (e.g. study risk of bias, inconsistency and imprecision). | YES |
| Interpretation | 10 | Provide a general interpretation of the results and important implications. | YES |
| **OTHER** | | |  |
| Funding | 11 | Specify the primary source of funding for the review. | NO |
| Registration | 12 | Provide the register name and registration number. | NA |

1. **Supplementary Figures:**

**Figure S1. Risk of bias (RoB) assessment for animal studies using the SYRCLE RoB tool.** (A) Representative summary Table for the risk of bias assessment. Green cells with ‘+’ designate low risk of bias, yellow cells with ‘?’ designate unclear risk of bias, and red cells with ‘-’ designate high risk of bias. (B) Representative summary of risk of bias analysis across studies.

**
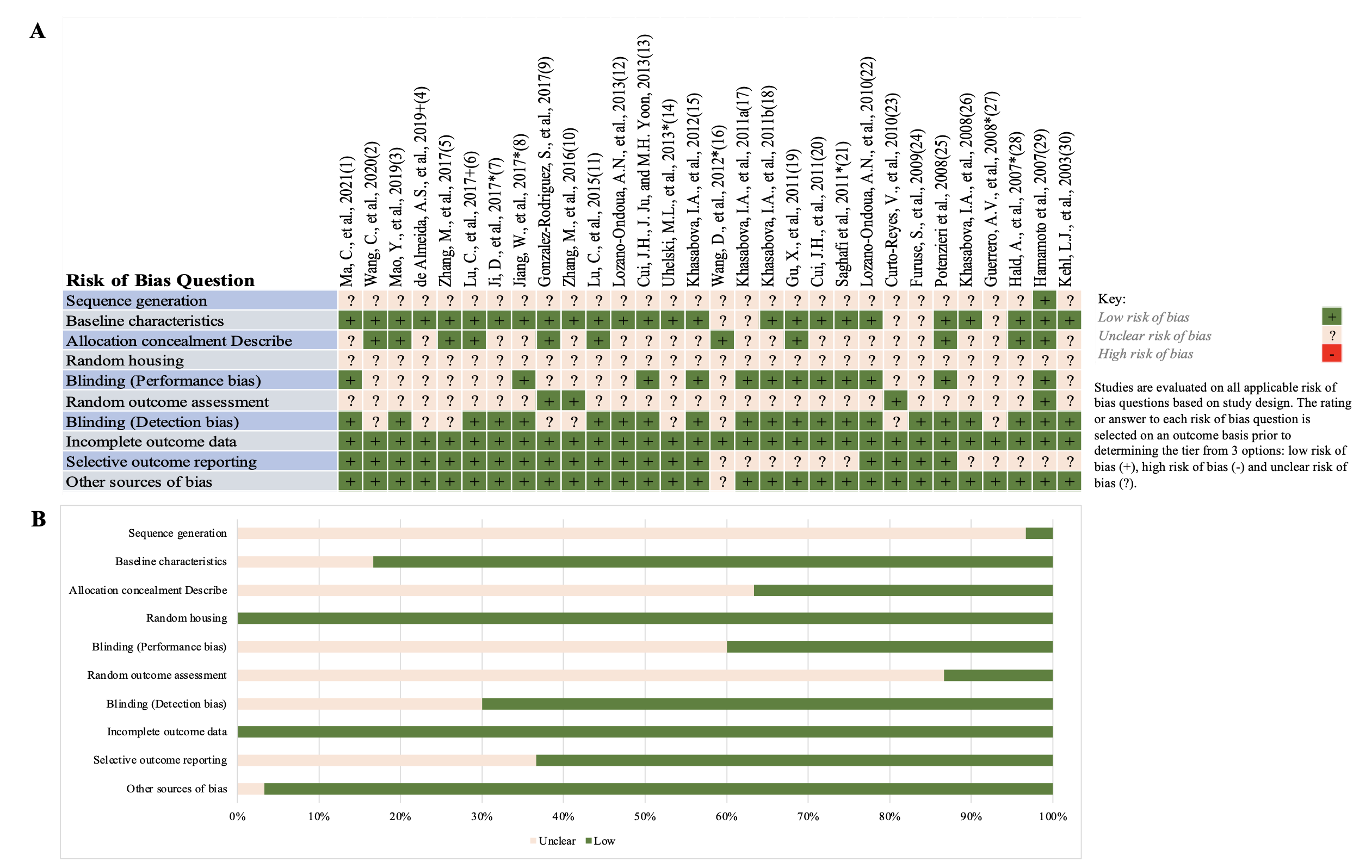
**

**Figure S2. Risk of bias (RoB) assessment for human studies using the revised Cochrane risk-of-bias tool (RoB 2) for randomized trials (RoB 2 tool)** Representative summary tables for the risk of bias assessment for randomized controlled trials (A) and crossover trials (B). Green cells with ‘+’ designate low risk of bias, yellow cells with ‘?’ designate some concerns, and red cells with ‘-’ designate high risk of bias. Representative summary of risk of bias analysis across randomized controlled trials (C) and crossover studies (D).

**
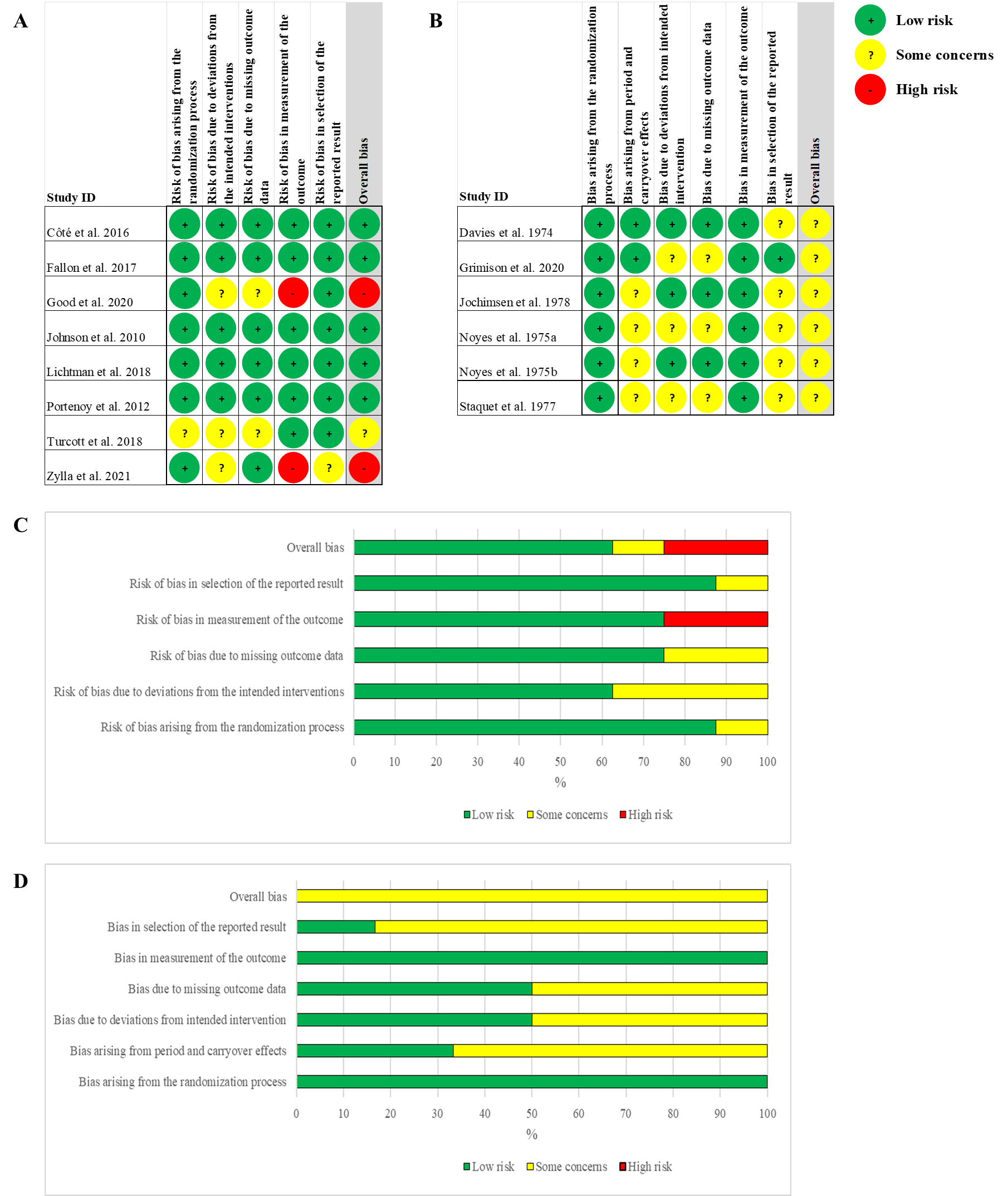
**

**Figure S3. The network of predicted associations for CB_1_, CB_2_ or CB_1/2_ in mouse (A), rat (B) and human (C) in STRING database.** Red line indicates the presence of fusion evidence, green line represents neighborhood evidence, blue line indicates cooccurrence evidence, purple line refers to experimental evidence, yellow line indicates text mining evidence, light blue line represents database evidence and black line refers to co-expression evidence.

**
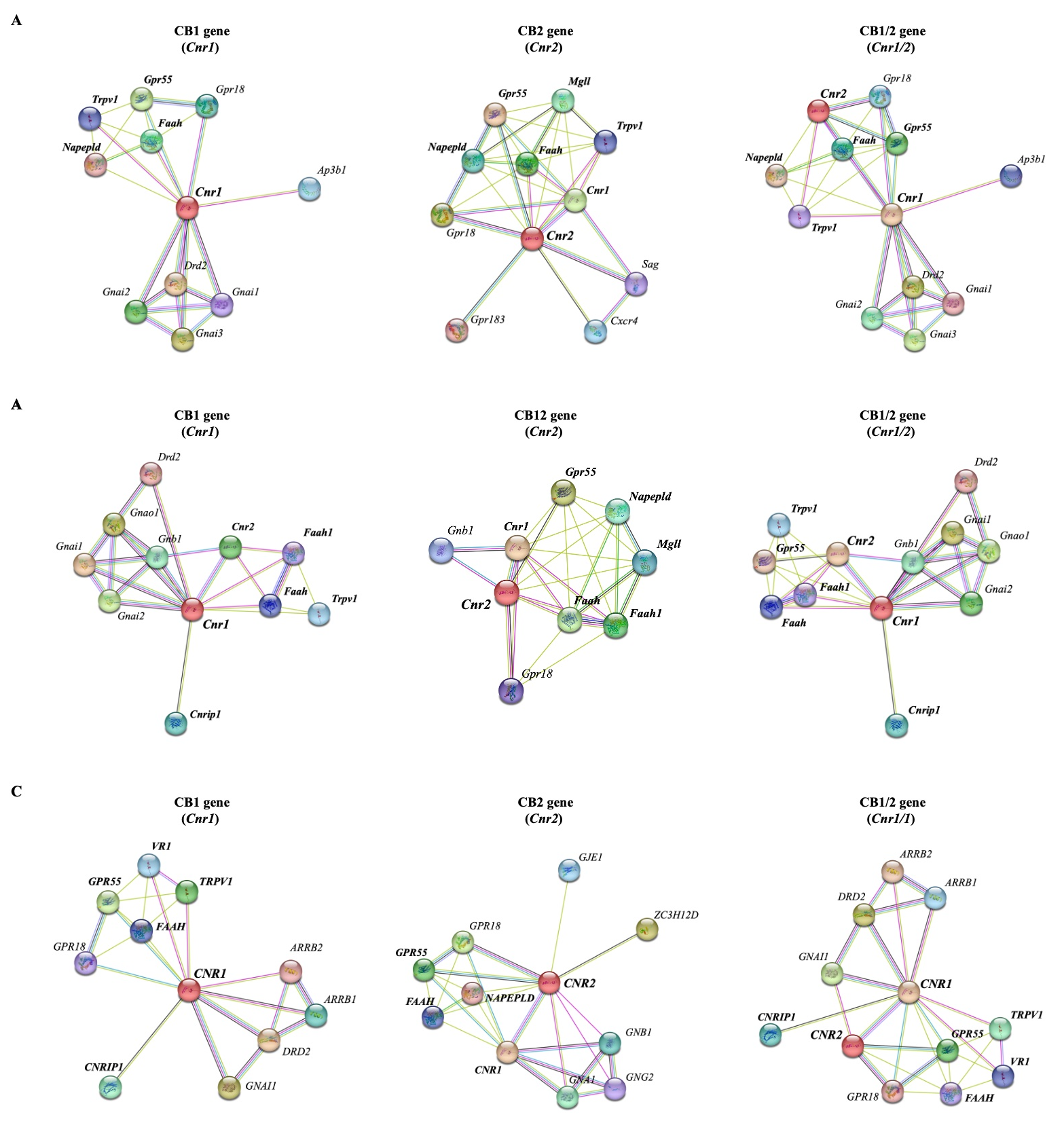
**

**Figure S4. Chemical structures of the mentioned cannabinoids.**

**
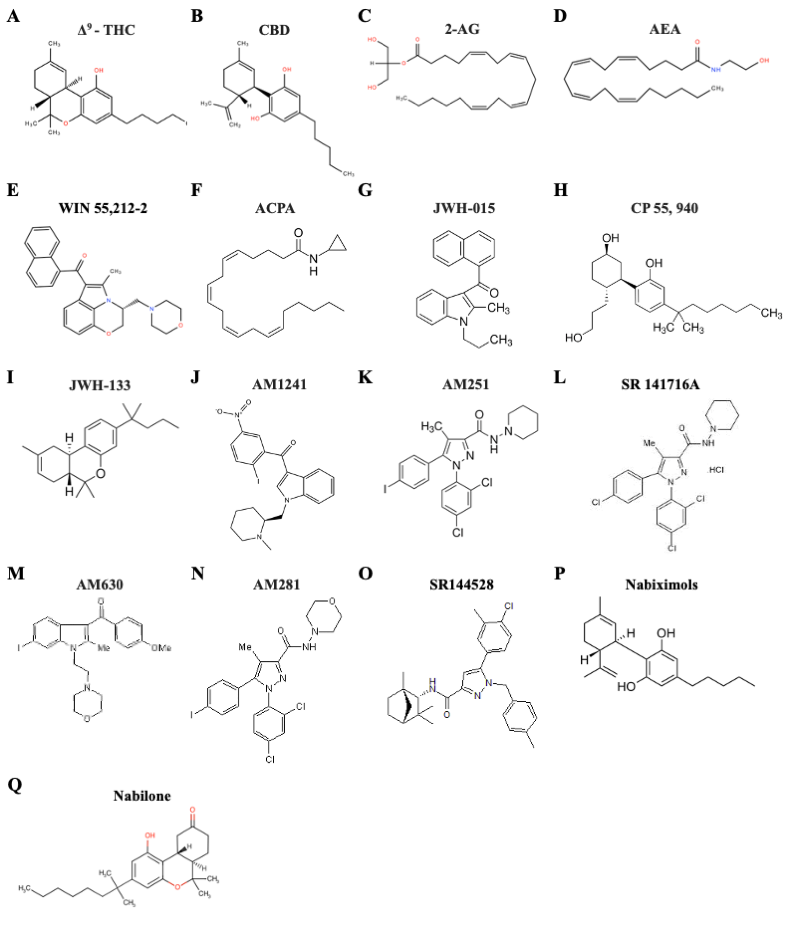
**

**3. Supplementary references:**

1 Ma, C. *et al.* Low-dose cannabinoid receptor 2 agonist induces microglial activation in a cancer pain-morphine tolerance rat model. *Life sciences* **264**, 118635 (2021). <https://doi.org:https://dx.doi.org/10.1016/j.lfs.2020.118635>

2 Wang, C. *et al.* Spinal cannabinoid receptor 2 activation reduces hypersensitivity associated with bone cancer pain and improves the integrity of the blood-spinal cord barrier. *Regional Anesthesia and Pain Medicine* **45**, 783-791 (2020). <https://doi.org:10.1136/rapm-2019-101262>

3 Mao, Y. *et al.* Cannabinoid receptor 2‑selective agonist JWH015 attenuates bone cancer pain through the amelioration of impaired autophagy flux induced by inflammatory mediators in the spinal cord. *Molecular Medicine Reports* **20**, 5100-5110 (2019). <https://doi.org:10.3892/mmr.2019.10772>

4 Zhang, M. *et al.* Effects of coadministration of low dose cannabinoid type 2 receptor agonist and morphine on vanilloid receptor 1 expression in a rat model of cancer pain. *Molecular medicine reports* **16**, 7025-7031 (2017). <https://doi.org:https://dx.doi.org/10.3892/mmr.2017.7479>

5 Lu, C. *et al.* A Single Intrathecal or Intraperitoneal Injection of CB2 Receptor Agonist Attenuates Bone Cancer Pain and Induces a Time-Dependent Modification of GRK2. *Cellular and Molecular Neurobiology* **37**, 101-109 (2017). <https://doi.org:10.1007/s10571-016-0349-0>

6 Ji, D., Liang, Z., Liu, G., Zhao, G. & Fang, J. Bufalin attenuates cancer-induced pain and bone destruction in a model of bone cancer. *Naunyn-Schmiedeberg's archives of pharmacology* **390**, 1211-1219 (2017). <https://doi.org:https://dx.doi.org/10.1007/s00210-017-1419-7>

7 Jiang, W., Wang, Y., Sun, W. & Zhang, M. Morin Suppresses Astrocyte Activation and Regulates Cytokine Release in Bone Cancer Pain Rat Models. *Phytotherapy research : PTR* **31**, 1298-1304 (2017). <https://doi.org:https://dx.doi.org/10.1002/ptr.5849>

8 Gonzalez-Rodriguez, S. *et al.* Synergistic combinations of the dual enkephalinase inhibitor PL265 given orally with various analgesic compounds acting on different targets, in a murine model of cancer-induced bone pain. *Scandinavian journal of pain* **14**, 25-38 (2017). <https://doi.org:https://dx.doi.org/10.1016/j.sjpain.2016.09.011>

9 Zhang, M. *et al.* Low-dose cannabinoid type 2 receptor agonist attenuates tolerance to repeated morphine administration via regulating μ-opioid receptor expression in walker 256 tumor-bearing rats. *Anesthesia and Analgesia* **122**, 1031-1037 (2016). <https://doi.org:10.1213/ANE.0000000000001129>

10 Lu, C. *et al.* Intrathecal Injection of JWH-015 Attenuates Bone Cancer Pain Via Time-Dependent Modification of Pro-inflammatory Cytokines Expression and Astrocytes Activity in Spinal Cord. *Inflammation* **38**, 1880-1890 (2015). <https://doi.org:10.1007/s10753-015-0168-3>

11 Lozano-Ondoua, A. N. *et al.* Disease modification of breast cancer-induced bone remodeling by cannabinoid 2 receptor agonists. *Journal of bone and mineral research : the official journal of the American Society for Bone and Mineral Research* **28**, 92-107 (2013). <https://doi.org:https://dx.doi.org/10.1002/jbmr.1732>

12 Cui, J. H., Ju, J. & Yoon, M. H. Pharmacology of cannabinoid receptor agonists and a cyclooxygenase-2 inhibitor in rat bone tumor pain. *Pharmacology* **92**, 150-157 (2013). <https://doi.org:https://dx.doi.org/10.1159/000354296>

13 Uhelski, M. L., Cain, D. M., Harding-Rose, C. & Simone, D. A. The non-selective cannabinoid receptor agonist WIN 55,212-2 attenuates responses of C-fiber nociceptors in a murine model of cancer pain. *Neuroscience* **247**, 84-94 (2013). <https://doi.org:https://dx.doi.org/10.1016/j.neuroscience.2013.05.003>

14 Wang, D. *et al.* [Role of cannabinoid 2 receptor in the development of bone cancer pain]. *Zhonghua yi xue za zhi* **92**, 440-443 (2012). <https://doi.org:https://dx.doi.org/10.3760/cma.j.issn.00376-2491-2012.07.003>

15 Khasabova, I. A. *et al.* CB1 and CB2 receptor agonists promote analgesia through synergy in a murine model of tumor pain. *BEHAVIOURAL PHARMACOLOGY* **22**, 607-616 (2011). <https://doi.org:10.1097/FBP.0b013e3283474a6d>

16 Khasabova, I. A., Chandiramani, A., Harding-Rose, C., Simone, D. A. & Seybold, V. S. Increasing 2-arachidonoyl glycerol signaling in the periphery attenuates mechanical hyperalgesia in a model of bone cancer pain. *Pharmacological research* **64**, 60-67 (2011). <https://doi.org:https://dx.doi.org/10.1016/j.phrs.2011.03.007>

17 Gu, X. *et al.* Intrathecal administration of the cannabinoid 2 receptor agonist JWH015 can attenuate cancer pain and decrease mRNA expression of the 2B subunit of N-methyl-D-aspartic acid. *Anesthesia and analgesia* **113**, 405-411 (2011). <https://doi.org:https://dx.doi.org/10.1213/ANE.0b013e31821d1062>

18 Cui, J. H. *et al.* Antinociceptive effect of intrathecal cannabinoid receptor agonist WIN 55,212-2 in a rat bone tumor pain model. *Neuroscience letters* **493**, 67-71 (2011). <https://doi.org:https://dx.doi.org/10.1016/j.neulet.2010.12.052>

19 Saghafi, N., Lam, D. K. & Schmidt, B. L. Cannabinoids attenuate cancer pain and proliferation in a mouse model. *Neuroscience letters* **488**, 247-251 (2011). <https://doi.org:https://dx.doi.org/10.1016/j.neulet.2010.11.039>

20 Lozano-Ondoua, A. N. *et al.* A cannabinoid 2 receptor agonist attenuates bone cancer-induced pain and bone loss. *Life sciences* **86**, 646-653 (2010). <https://doi.org:https://dx.doi.org/10.1016/j.lfs.2010.02.014>

21 Curto-Reyes, V., Llames, S., Hidalgo, A., Menéndez, L. & Baamonde, A. Spinal and peripheral analgesic effects of the CB2 cannabinoid receptor agonist AM1241 in two models of bone cancer-induced pain. *British Journal of Pharmacology* **160**, 561-573 (2010). <https://doi.org:10.1111/j.1476-5381.2009.00629.x>

22 Furuse, S. *et al.* Reduction of Bone Cancer Pain by Activation of Spinal Cannabinoid Receptor 1 and Its Expression in the Superficial Dorsal Horn of the Spinal Cord in a Murine Model of Bone Cancer Pain. *ANESTHESIOLOGY* **111**, 173-186 (2009). <https://doi.org:10.1097/ALN.0b013e3181a51e0d>

23 Potenzieri, C., Harding-Rose, C. & Simone, D. A. The cannabinoid receptor agonist, WIN 55, 212-2, attenuates tumor-evoked hyperalgesia through peripheral mechanisms. *Brain research* **1215**, 69-75 (2008). <https://doi.org:https://dx.doi.org/10.1016/j.brainres.2008.03.063>

24 Khasabova, I. A. *et al.* A decrease in anandamide signaling contributes to the maintenance of cutaneous mechanical hyperalgesia in a model of bone cancer pain. *The Journal of neuroscience : the official journal of the Society for Neuroscience* **28**, 11141-11152 (2008). <https://doi.org:https://dx.doi.org/10.1523/JNEUROSCI.2847-08.2008>

25 Guerrero, A. V., Quang, P., Dekker, N., Jordan, R. C. K. & Schmidt, B. L. Peripheral cannabinoids attenuate carcinoma-induced nociception in mice. *Neuroscience letters* **433**, 77-81 (2008). <https://doi.org:https://dx.doi.org/10.1016/j.neulet.2007.12.053>

26 Hald, A. *et al.* Differential effects of repeated low dose treatment with the cannabinoid agonist WIN 55,212-2 in experimental models of bone cancer pain and neuropathic pain. *Pharmacology, biochemistry, and behavior* **91**, 38-46 (2008). <https://doi.org:https://dx.doi.org/10.1016/j.pbb.2008.04.021>

27 Hamamoto, D. T., Giridharagopalan, S. & Simone, D. A. Acute and chronic administration of the cannabinoid receptor agonist CP 55,940 attenuates tumor-evoked hyperalgesia. *European journal of pharmacology* **558**, 73-87 (2007).

28 Kehl, L. J. *et al.* A cannabinoid agonist differentially attenuates deep tissue hyperalgesia in animal models of cancer and inflammatory muscle pain. *Pain* **103**, 175-186 (2003).

29 de Almeida, A. S. *et al.* Characterization of Cancer-Induced Nociception in a Murine Model of Breast Carcinoma. *Cell Mol Neurobiol* **39**, 605-617 (2019). <https://doi.org:10.1007/s10571-019-00666-8>

30 Davies, B. H., Weatherstone, R. M., Graham, J. D. & Griffiths, R. D. A pilot study of orally administered Δ(1)-trans-tetrahydrocannabinol in the management of patients undergoing radiotherapy for carcinoma of the bronchus. *Br J Clin Pharmacol* **1**, 301-306 (1974). <https://doi.org:10.1111/j.1365-2125.1974.tb00257.x>

31 Grimison, P. *et al.* Oral THC:CBD cannabis extract for refractory chemotherapy-induced nausea and vomiting: a randomised, placebo-controlled, phase II crossover trial. *Ann Oncol* **31**, 1553-1560 (2020). <https://doi.org:10.1016/j.annonc.2020.07.020>

32 Jochimsen, P. R., Lawton, R. L., VerSteeg, K. & Noyes, R., Jr. Effect of benzopyranoperidine, a delta-9-THC congener, on pain. *Clin Pharmacol Ther* **24**, 223-227 (1978). <https://doi.org:10.1002/cpt1978242223>

33 Noyes, R., Jr., Brunk, S. F., Avery, D. A. & Canter, A. C. The analgesic properties of delta-9-tetrahydrocannabinol and codeine. *Clin Pharmacol Ther* **18**, 84-89 (1975). <https://doi.org:10.1002/cpt197518184>

34 Noyes, R., Jr., Brunk, S. F., Baram, D. A. & Canter, A. Analgesic effect of delta-9-tetrahydrocannabinol. *J Clin Pharmacol* **15**, 139-143 (1975). <https://doi.org:10.1002/j.1552-4604.1975.tb02348.x>

35 Staquet, M., Gantt, C. & Machin, D. Effect of a nitrogen analog of tetrahydrocannabinol on cancer pain. *Clin Pharmacol Ther* **23**, 397-401 (1978). <https://doi.org:10.1002/cpt1978234397>

36 Fallon, M. T. *et al.* Sativex oromucosal spray as adjunctive therapy in advanced cancer patients with chronic pain unalleviated by optimized opioid therapy: two double-blind, randomized, placebo-controlled phase 3 studies. *Br J Pain* **11**, 119-133 (2017). <https://doi.org:10.1177/2049463717710042>

37 Johnson, J. R. *et al.* Multicenter, double-blind, randomized, placebo-controlled, parallel-group study of the efficacy, safety, and tolerability of THC:CBD extract and THC extract in patients with intractable cancer-related pain. *J Pain Symptom Manage* **39**, 167-179 (2010). <https://doi.org:10.1016/j.jpainsymman.2009.06.008>

38 Turcott, J. G. *et al.* The effect of nabilone on appetite, nutritional status, and quality of life in lung cancer patients: a randomized, double-blind clinical trial. *Support Care Cancer* **26**, 3029-3038 (2018). <https://doi.org:10.1007/s00520-018-4154-9>

39 Côté, M., Trudel, M., Wang, C. & Fortin, A. Improving Quality of Life With Nabilone During Radiotherapy Treatments for Head and Neck Cancers: A Randomized Double-Blind Placebo-Controlled Trial. *Annals of Otology, Rhinology & Laryngology* **125**, 317-324 (2015). <https://doi.org:10.1177/0003489415612801>

40 Lichtman, A. H. *et al.* Results of a Double-Blind, Randomized, Placebo-Controlled Study of Nabiximols Oromucosal Spray as an Adjunctive Therapy in Advanced Cancer Patients with Chronic Uncontrolled Pain. *J Pain Symptom Manage* **55**, 179-188.e171 (2018). <https://doi.org:10.1016/j.jpainsymman.2017.09.001>

41 Portenoy, R. K. *et al.* Nabiximols for opioid-treated cancer patients with poorly-controlled chronic pain: a randomized, placebo-controlled, graded-dose trial. *J Pain* **13**, 438-449 (2012). <https://doi.org:10.1016/j.jpain.2012.01.003>

42 Zylla, D. M. *et al.* A randomized trial of medical cannabis in patients with stage IV cancers to assess feasibility, dose requirements, impact on pain and opioid use, safety, and overall patient satisfaction. *Support Care Cancer* **29**, 7471-7478 (2021). <https://doi.org:10.1007/s00520-021-06301-x>

43 Good, P. D., Greer, R. M., Huggett, G. E. & Hardy, J. R. An Open-Label Pilot Study Testing the Feasibility of Assessing Total Symptom Burden in Trials of Cannabinoid Medications in Palliative Care. *J Palliat Med* **23**, 650-655 (2020). <https://doi.org:10.1089/jpm.2019.0540>

44 Tavhare, S. D., Acharya, R., Reddy, R. G. & Dhiman, K. S. Management of chronic pain with Jalaprakshalana (water-wash) Shodhita (processed) Bhanga (Cannabis sativa L.) in cancer patients with deprived quality of life: An open-label single arm clinical trial. *Ayu* **40**, 34-43 (2019). <https://doi.org:10.4103/ayu.AYU_43_19>

45 Bar-Sela, G., Zalman, D., Semenysty, V. & Ballan, E. The Effects of Dosage-Controlled Cannabis Capsules on Cancer-Related Cachexia and Anorexia Syndrome in Advanced Cancer Patients: Pilot Study. *Integr Cancer Ther* **18**, 1534735419881498 (2019). <https://doi.org:10.1177/1534735419881498>

46 Bar-Lev Schleider, L. *et al.* Prospective analysis of safety and efficacy of medical cannabis in large unselected population of patients with cancer. *Eur J Intern Med* **49**, 37-43 (2018). <https://doi.org:10.1016/j.ejim.2018.01.023>

47 Bar-Sela, G. *et al.* The medical necessity for medicinal cannabis: prospective, observational study evaluating the treatment in cancer patients on supportive or palliative care. *Evid Based Complement Alternat Med* **2013**, 510392 (2013). <https://doi.org:10.1155/2013/510392>

48 Aviram, J. *et al.* Short-Term Medical Cannabis Treatment Regimens Produced Beneficial Effects among Palliative Cancer Patients. *Pharmaceuticals (Basel)* **13** (2020). <https://doi.org:10.3390/ph13120435>

49 Kasvis, P., Canac-Marquis, M., Aprikian, S., Vigano, M. & Vigano, A. Sex differences exist in the perceived relief of cancer symptoms with medical cannabis: results from the Quebec Cannabis Registry. *Support Care Cancer* **30**, 7863-7871 (2022). <https://doi.org:10.1007/s00520-022-07193-1>

50 Meghani, S. H. *et al.* Impact of Cannabis Use on Least Pain Scores Among African American and White Patients with Cancer Pain: A Moderation Analysis. *J Pain Res* **14**, 3493-3502 (2021). <https://doi.org:10.2147/jpr.S332447>

51 Johnson, J. R., Lossignol, D., Burnell-Nugent, M. & Fallon, M. T. An open-label extension study to investigate the long-term safety and tolerability of THC/CBD oromucosal spray and oromucosal THC spray in patients with terminal cancer-related pain refractory to strong opioid analgesics. *J Pain Symptom Manage* **46**, 207-218 (2013). <https://doi.org:10.1016/j.jpainsymman.2012.07.014>

52 Raghunathan, N. J. *et al.* In the weeds: a retrospective study of patient interest in and experience with cannabis at a cancer center. *Supportive Care in Cancer* **30**, 7491-7497 (2022). <https://doi.org:10.1007/s00520-022-07170-8>

53 Wiseman, L. K., Mahu, I. T. & Mukhida, K. The Effect of Preoperative Cannabis Use on Postoperative Pain Following Gynaecologic Oncology Surgery. *J Obstet Gynaecol Can* **44**, 750-756 (2022). <https://doi.org:10.1016/j.jogc.2022.01.018>

54 Pawasarat, I. M. *et al.* The Efficacy of Medical Marijuana in the Treatment of Cancer-Related Pain. *J Palliat Med* **23**, 809-816 (2020). <https://doi.org:10.1089/jpm.2019.0374>

55 Donovan, K. A. *et al.* Cannabis Use in Young Adult Cancer Patients. *J Adolesc Young Adult Oncol* **9**, 30-35 (2020). <https://doi.org:10.1089/jayao.2019.0039>

56 Donovan, K. A. *et al.* Relationship of Cannabis Use to Patient-Reported Symptoms in Cancer Patients Seeking Supportive/Palliative Care. *J Palliat Med* **22**, 1191-1195 (2019). <https://doi.org:10.1089/jpm.2018.0533>

57 Nathan, R. *et al.* Assessing Efficacy and Use Patterns of Medical Cannabis for Symptom Management in Elderly Cancer Patients. *Am J Hosp Palliat Care*, 10499091221110217 (2022). <https://doi.org:10.1177/10499091221110217>

58 Anderson, S. P., Zylla, D. M., McGriff, D. M. & Arneson, T. J. Impact of Medical Cannabis on Patient-Reported Symptoms for Patients With Cancer Enrolled in Minnesota's Medical Cannabis Program. *J Oncol Pract* **15**, e338-e345 (2019). <https://doi.org:10.1200/jop.18.00562>

59 Waissengrin, B., Urban, D., Leshem, Y., Garty, M. & Wolf, I. Patterns of use of medical cannabis among Israeli cancer patients: a single institution experience. *J Pain Symptom Manage* **49**, 223-230 (2015). <https://doi.org:10.1016/j.jpainsymman.2014.05.018>

60 Ofir, R., Bar-Sela, G., Weyl Ben-Arush, M. & Postovsky, S. Medical marijuana use for pediatric oncology patients: single institution experience. *Pediatr Hematol Oncol* **36**, 255-266 (2019). <https://doi.org:10.1080/08880018.2019.1630537>

61 Calcaterra, S. L. *et al.* A population-based survey to assess the association between cannabis and quality of life among colorectal cancer survivors. *BMC Cancer* **20**, 373 (2020). <https://doi.org:10.1186/s12885-020-06887-1>

62 Chapman, S. *et al.* Medical cannabis in pediatric oncology: a survey of patients and caregivers. *Support Care Cancer* **29**, 6589-6594 (2021). <https://doi.org:10.1007/s00520-021-06202-z>

63 Webster, E. M. *et al.* Prescribed medical cannabis in women with gynecologic malignancies: A single-institution survey-based study. *Gynecol Oncol Rep* **34**, 100667 (2020). <https://doi.org:10.1016/j.gore.2020.100667>

64 Elliott, D. A., Nabavizadeh, N., Romer, J. L., Chen, Y. & Holland, J. M. Medical marijuana use in head and neck squamous cell carcinoma patients treated with radiotherapy. *Supportive Care in Cancer* **24**, 3517-3524 (2016). <https://doi.org:10.1007/s00520-016-3180-8>
